# Recommendations for reporting ion mobility Mass Spectrometry measurements

**DOI:** 10.1002/mas.21585

**Published:** 2019-02-01

**Authors:** Valérie Gabelica, Alexandre A. Shvartsburg, Carlos Afonso, Perdita Barran, Justin L.P. Benesch, Christian Bleiholder, Michael T. Bowers, Aivett Bilbao, Matthew F. Bush, J. Larry Campbell, Iain D.G. Campuzano, Tim Causon, Brian H. Clowers, Colin S. Creaser, Edwin De Pauw, Johann Far, Francisco Fernandez‐Lima, John C. Fjeldsted, Kevin Giles, Michael Groessl, Christopher J. Hogan, Stephan Hann, Hugh I. Kim, Ruwan T. Kurulugama, Jody C. May, John A. McLean, Kevin Pagel, Keith Richardson, Mark E. Ridgeway, Frédéric Rosu, Frank Sobott, Konstantinos Thalassinos, Stephen J. Valentine, Thomas Wyttenbach

**Affiliations:** ^1^ University of Bordeaux, INSERM and CNRS, ARNA Laboratory, IECB site 2 rue Robert Escarpit, 33600 Pessac France; ^2^ Department of Chemistry Wichita State University 1845 Fairmount St. Wichita Kansas 67620; ^3^ Université de Rouen Mont‐Saint‐Aignan France; ^4^ Michael Barber Centre for Collaborative Mass Spectrometry Manchester Institute for Biotechnology, University of Manchester Manchester UK; ^5^ Department of Chemistry, Chemistry Research Laboratory University of Oxford, Mansfield Road, OX1 3TA Oxford UK; ^6^ Department of Chemistry and Biochemistry Florida State University Tallahassee Florida 32311; ^7^ University of California Santa Barbara California; ^8^ Biological Sciences Division Pacific Northwest National Laboratory Richland Washington; ^9^ Department of Chemistry University of Washington Seattle Washington; ^10^ SCIEX, Concord Ontario Canada; ^11^ Amgen Discovery Research Thousand Oaks California; ^12^ University of Natural Resources and Life Sciences (BOKU) Department of Chemistry, Division of Analytical Chemistry Vienna Austria; ^13^ Department of Chemistry Washington State University Pullman Washington; ^14^ Centre for Analytical Science Department of Chemistry, Loughborough University Loughborough UK; ^15^ Laboratoire de spectrométrie de masse (L.S.M.) − Molecular Systems Université de Liège Liège Belgium; ^16^ Department of Chemistry and Biochemistry Florida International University Miami Florida; ^17^ Agilent Technologies Santa Clara California; ^18^ Waters Corporation Wilmslow UK; ^19^ Department of Nephrology and Hypertension and Department of BioMedical Research Inselspital, Bern University Hospital, University of Bern, Switzerland and Tofwerk Thun Switzerland; ^20^ Department of Mechanical Engineering University of Minnesota Minneapolis Minnesota; ^21^ Department of Chemistry Korea University Seoul Korea; ^22^ Department of Chemistry Center for Innovative Technology, Vanderbilt University Nashville Tennessee; ^23^ Freie Universitaet Berlin Institute for Chemistry and Biochemistry Berlin Germany; ^24^ Bruker Daltonics Billerica Massachusetts; ^25^ CNRS, INSERM and University of Bordeaux Institut Européen de Chimie et Biologie Pessac France; ^26^ Antwerp University Biomolecular & Analytical Mass Spectrometry Antwerp Belgium; ^27^ Astbury Centre for Structural Molecular Biology University of Leeds Leeds UK; ^28^ School of Molecular and Cellular Biology University of Leeds Leeds UK; ^29^ Institute of Structural and Molecular Biology, Division of Biosciences University College London London WC1E 6BT UK; ^30^ United Kingdom and Institute of Structural and Molecular Biology Department of Biological Sciences, Birkbeck College, University of London London WC1E 7HX UK; ^31^ C. Eugene Bennett Department of Chemistry West Virginia University Morgantown West Virginia

## Abstract

Here we present a guide to ion mobility mass spectrometry experiments, which covers both linear and nonlinear methods: what is measured, how the measurements are done, and how to report the results, including the uncertainties of mobility and collision cross section values. The guide aims to clarify some possibly confusing concepts, and the reporting recommendations should help researchers, authors and reviewers to contribute comprehensive reports, so that the ion mobility data can be reused more confidently. Starting from the concept of the definition of the measurand, we emphasize that (i) mobility values (*K*_0_) depend intrinsically on ion structure, the nature of the bath gas, temperature, and *E*/*N*; (ii) ion mobility does not measure molecular surfaces directly, but collision cross section (CCS) values are derived from mobility values using a physical model; (iii) methods relying on calibration are empirical (and thus may provide method‐dependent results) only if the gas nature, temperature or *E*/*N* cannot match those of the primary method. Our analysis highlights the urgency of a community effort toward establishing primary standards and reference materials for ion mobility, and provides recommendations to do so. © 2019 The Authors. *Mass Spectrometry Reviews* Published by Wiley Periodicals, Inc.

## I. INTRODUCTION

The ion mobility spectrometry (IMS) technique measures ions drift through a region filled with a buffer gas, under the influence of an electric field. IMS was first reported in 1898 (Zeleny, 1898), approximately 15 years before mass spectrometry (MS) (Thompson, 1912), with both analytical methods originating in the Cavendish laboratory at Cambridge University. The coupling of IMS to MS for analytical purposes dates back to 1970 (Cohen & Karasek, 1970). In IMS‐MS, the ions are detected at the end, but are successively separated by two different principles: IMS then MS. IMS is often presented as an additional dimension of separation added to MS. The abbreviation “IM‐MS” (ion mobility mass spectrometry) is also commonly encountered; it is equivalent to “IMS‐MS” and both are acceptable.

In 2006, the availability of IM‐MS instrumentation from a major MS manufacturer marked the onset of a growing use of IM for the separation, identification, and structural characterization of analytes across diverse fields of science. These include: ionic clusters (Bowers et al., 1993; Von Helden et al., 1993; Dugourd et al., 1998; Hudgins et al., 1999; Gilb et al., 2002), catalysts (Czerwinska et al., 2016; Greisch et al., 2018), supramolecular complexes (Li et al., 2011; Ujma et al., 2012; Warzok et al., 2018; Wollschlager et al., 2018), small organic molecules including drugs (Campuzano et al., 2012; Hines et al., 2017), lipids (Hinz, Liggi, & Griffin, 2018; Zheng, Smith, & Baker, 2018), and other metabolites (Far et al., 2014; Mairinger, Causon, & Hann, 2018; Zhang et al., 2018), glycans (Hofmann & Pagel, 2017; Chen, Glover, & Li, 2018; Morrison & Clowers, 2018), peptides (Valentine et al., 1998; Wu et al., 2000; Harvey, Macphee, & Barran, 2011), proteins (Clemmer, Hudgins, & Jarrold, 1995; Jarrold, 1999; McLean et al., 2005), synthetic polymers (Trimpin & Clemmer, 2008; Morsa et al., 2014; Wesdemiotis, 2017), biomolecules (Clemmer & Jarrold, 1997; Fenn & McLean, 2008) and biomolecular complexes (Benesch & Ruotolo, 2011; Liko et al., 2016; Ben‐Nissan & Sharon, 2018). However, as IM‐MS instruments now commercially available from different manufacturers operate according to different principles of ion mobility separation (May & McLean, 2015), this introduces complexity—and potentially confusion—among practitioners and readers. The increasing rate at which IM data are being generated has established the need for a community‐coordinated set of recommendations to report IM‐MS results. The present article is a first step to unify the community in this respect.

In IM separations, mobility values (*K* or *K*_0_) can be used akin to retention times in chromatographic analyses (with *K*_0_ being more invariant). Today, standalone IMS is widely used for defense (Eiceman & Stone, 2004), security (Ewing et al., 2001) and environmental (Zheng et al., 2017) applications. Further, thanks to the availability of IM‐MS instruments, scientists active in the fields of “omics” have also begun to adopt IMS as an additional separation dimension (May, Gant‐Branum, & McLean, 2016; Barran & Baker, 2018). Furthermore, mobility values, or a derived property such as the ion‐neutral collision cross section (CCS) can be calculated from structural models of ions. Ion mobility measurements can hence serve a two‐fold purpose; they can add a separation dimension that is partly orthogonal to mass spectrometry, and can be used for structural elucidation. Structural inferences based on comparing experimental CCS values with those calculated from 3D models have become increasingly prominent in structural biology (Politis et al., 2010; Konijnenberg, Butterer, & Sobott, 2013; Thalassinos et al., 2013; Marklund et al., 2015; Ben‐Nissan & Sharon, 2018), structural chemistry (Ujma et al., 2012; Surman et al., 2016) and physical chemistry (Wyttenbach et al., 2014). For these reasons, we devoted special attention to define *K*_0_ and IM‐derived *CCS* values, and to clarify what influences these quantities. There are other methods than ion mobility spectrometry to determine experimental collision cross sections in a more direct way, for example, by monitoring the loss of ion signal due to collisions (Covey & Douglas, 1993; Chen, Collings, & Douglas, 1997; Anupriya, Jones, & Dearden, 2016; Anupriya et al., 2017; Dziekonski et al., 2017; Elliott et al., 2017; Sanders et al., 2018). These methods to determine collision cross sections are however not based on ion mobility measurements, and will not be covered here.

Ion mobility spectrometry is a measurement science, and should thus be performed and reported according to the internationally recognized best practices in metrology, including traceability to the international systems of units, use of standards, and evaluation of measurement uncertainty. This critical review devoteds particular attention to the uncertainty (JCGM, 2008; EURACHEM/CITAC, 2012) associated with *K*_0_ and *CCS* values. Over the years, several groups have published tables of CCS values (Wyttenbach, Von Helden, & Bowers, 1996; Hoaglund et al., 1997; Shelimov et al., 1997; Valentine et al., 1997; Valentine, Counterman, & Clemmer, 1997; Counterman et al., 1998; Hoaglund et al., 1998; Wyttenbach, Bushnell, & Bowers, 1998; Henderson et al., 1999,1999; Valentine, Counterman, & Clemmer, 1999; Fenn et al., 2009; Bush et al., 2010; Bush, Campuzano, & Robinson, 2012; Campuzano et al., 2012; Salbo et al., 2012; Forsythe et al., 2015; May, Morris, & McLean, 2017), which are widely used as input values for calibrating instruments from which CCS values cannot be obtained directly. One significant source of confusion is that it is difficult to interpret differences in output values obtained on different instrumental platforms or when using different sets of calibrants, or different sets of values for the same calibrant. To establish whether differences are within the uncertainty of the measurement, or have a physical meaning (e.g., temperature or field effects) or a chemical meaning (e.g., different ion structures), one must first know the uncertainty associated with the calibrant values. Unfortunately, the lack of a common approach in reporting all details associated with values makes it difficult to establish the appropriate level of confidence in each value. Here we used international guidelines (Guideline to the expression of Uncertainty in Measurement or “GUM” (JCGM, 2008) and Quantifying Uncertainty in Analytical Measurement or “QUAM” (EURACHEM/CITAC, 2012)) to provide recommendations on how to report values and their associated uncertainty.

Another objective of this document is to clarify the data reporting across different instrumental platforms, to harmonize them and enable cross‐platform comparisons. The metrology considerations will lead us to underline a few “do's and don'ts,” but besides these, the recommendations will focus on *how to report* the data, rather than on how to obtain and treat the data. For the latter, consult the primary literature and reviews cited throughout the paper. We will however outline the scientific rationale that underlies the recommendations, and should underlie any future effort toward establishing minimum information standards, data formats which would update the JCAMP‐DX format proposed for standalone IM (Baumbach et al., 2001), and shared databases of experimentally derived values related to IM. Finally, we aim to guide authors, readers and reviewers on how to prepare comprehensive supporting information for IM‐MS reports.

## II. DEFINITIONS

The present guidelines are intended to be applied primarily to the determination of ion mobility values and of CCS values from IM‐MS experiments. It should be noted that there are other ways to determine collision cross sections, for example, using single‐collision scattering experiments, but that the definition of these collision cross sections is slightly different (see below). The present recommendations focus exclusively on IM experiments, wherein ions are directed by an electric field and experience many collisions with a background gas.

### A. Definition of the Measurand in Ion Mobility

Incomplete definition of the measurand (i.e., what is measured) is itself a source of uncertainty. Unlike mass, which is a unique property of a molecule, an ion's mobility and the ion‐neutral CCS depend not only on the composition and structure of the analyte, but also on other factors. For example, “the CCS of aniline” is vague and does not fully convey the context and conditions under which a measurement is made, such that many values can match this definition of the measurand. In contrast “the ion‐neutral CCS determined from the position of the *apex* of the most intense mobility peak of the [M + H]^+^ ion generated by electrospray ionization of aniline prepared at 50 ppm in a 49.5/49.5/1 (v:v) water/acetonitrile/formic acid solution in the softest possible source conditions (sampling cone of 10 V on instrument X), and measured in 4 mBar nitrogen drift gas at 298 K under an electric field of 13 V/cm” is more precise and useful for interpreting the reported value. For aniline (Attygalle, Xia, & Pavlov, 2017) and other small molecules that can form protonation isomers (tautomers), all these details matter (Steill & Oomens, 2009; Warnke et al., 2015; Boschmans et al., 2016; Xia & Attygalle, 2016; Xia & Attygalle, 2017). However, such textual description would be unpractical to implement in papers reporting ion mobility mass spectrometry measurements. We will thus provide here a concise notation, and a list of relevant parameters to report in the supplementary information of papers.

First, this example highlights that the property being measured is not an absolute, constant attribute of the analyte, but refers to a population of ions coming from a sample containing the analyte. The whole chain of events from the original sample to ion detection could potentially influence the result. These guidelines also recommend documenting the sample preparation, ion preparation (ion formation and desolvation/declustering, typically done in the source region of the spectrometer), ion transfer conditions (to account for all post‐ionization transformations due to ion‐molecule reactions and/or to ion activation), ion packet preparation before introduction into the IM device, and ion transfer conditions from the IM device to the detector at which the arrival time is measured.

Second, the measurement is not made on a single ion, but on an ensemble (population) of ions. Depending on the ion preparation, on the temperature in the IMS device and on the time spent in the device, the mobility and CCS properties of the ensemble can vary. The arrival time distribution (ATD) conveys information on how homogeneous or heterogeneous the ensemble is. Section II.B further elaborates on the importance of ATDs for the definition of the measurand.

Finally, the gas composition, temperature, pressure and field strength also influence the mobility of an ion, as explained in sections II.C and II.D. Table 1 clarifies the parameters that should be part of the definition of the measurand.

**Table 1:**
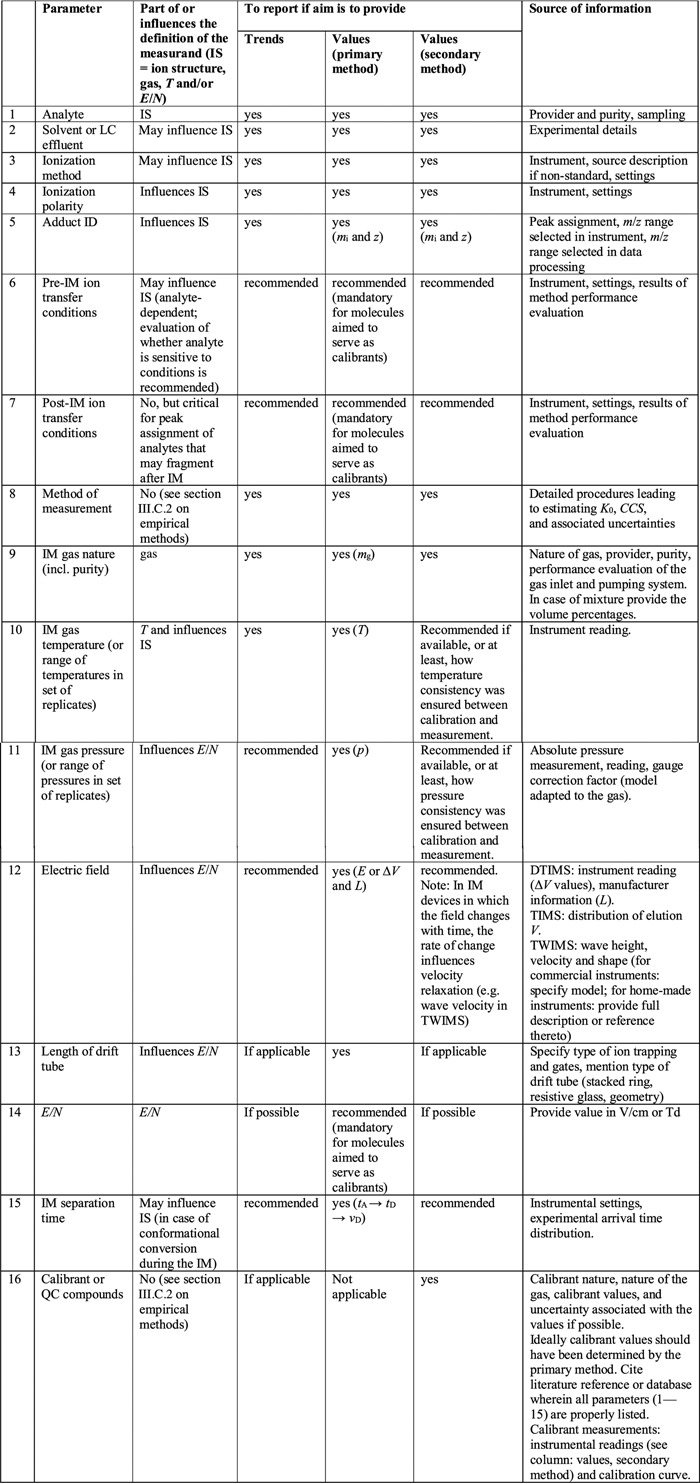
List of experimental parameters to be reported for an IM‐MS measurement

### B. Arrival Time Distributions (ATD)

Each ion takes a defined time to traverse the mobility region and, because the measurement is performed on a population of ions, a distribution of arrival times is obtained. The “arrival time distribution” (ATD) is defined as a 2D graph of the number of counts detected as a function of the time at which they arrive at a specific location in the instrument (comprising a drift time plus a time spent outside the drift region). Drift tube and traveling wave IM devices are time dispersive. In scanning devices (e.g., TIMS and DMA), a voltage is ramped as a function of time and the analyzer primarily measures the number of ion counts as a function of a time or scan number. We define ATDs in this broader sense. The term “mobilogram” is sometimes encountered in the literature reporting TWIMS data; but the terminology is not widely adopted and “arrival time distribution” or “ion mobility spectrum” should be preferred.

For a homogeneous population of ions, the width of ATD is primarily controlled by ion diffusion, Coulomb repulsion (space charge), the (temporal or spatial) width of ion injection pulse, the (temporal or spatial) width of detector acquisition window, and electric field homogeneity. This nominal width can be predicted theoretically (Siems et al., 1994; Wyttenbach, Von Helden, & Bowers, 1996; May et al., 2015), or determined experimentally from structurally rigid (e.g., C_60_) or homogeneous ions (Jeanne Dit Fouque et al., 2015; Kune, Far, & De Pauw, 2016), although this can be hard to achieve for large ions. However, the experimental ATD can be broader than predicted. Provided that the contribution of instrumental factors to the ATD has been properly established, additional information may thus be inferred from ATD widths that significantly deviate from the ideal. One reason can be that the ion population comprises multiple structures (in the broad sense, i.e., different shapes, different charge isomers, and/or different charges), each having different mobility and CCS values. In some cases, multiple peaks are discernable. Alternatively, the ATD width can be increased by space charge (in instances where the ion concentration is sufficiently high) as well as by structural dynamics and reactions of the ions during transit in the gas phase.

If the measurand is defined as the entire population of ions generated from the sample in given conditions, if the peak is broader than predicted for a population of ions with a single structure, then the distribution of values satisfying this definition of the measurand is also broad. For proteins, the full‐width at half‐maximum of the ATDs can be 10% of the centroid value (Uetrecht et al., 2010; Laszlo, Munger, & Bush, 2016), and up to 20% for intrinsically disordered proteins (Beveridge et al., 2014). However, if the measurand is defined as “the mobility or CCS associated with the centroid of the ATD of the ion population” (or any other precisely defined attribute of the distribution), the uncertainty on that value will be smaller.

The present recommendations for reporting mobility and CCS values, and their associated uncertainty, will here focus on reporting the values associated with the centroid of each distinguishable peak of the ATD. However, the above examples illustrate that, for structural characterization, both the representative value and the shape and width of the ATD are worth reporting. We will provide specific guidance accordingly.

### C. Mobility *K* and Reduced Mobility *K*_0_

In a device filled with a homogeneous gas and under the influence of a weak electric field *E* (*E* = Δ*V*/*L*), the ion's mobility (*K*) is defined as the ratio between the steady‐state net ion/gas relative velocity (*v*_d_ = *L*/*t*_d_) and the applied electric field *E* (Equation (1)).

(1) $$K=\frac{v_{d}}{E}$$

The mobility *K* provides information on the range of ion‐neutral interactions experienced by an ion as it traverses through a buffer gas. In a highly simplified definition, *K* thus depends on the collision frequency of the ion with gas molecules. This frequency depends on the gas number‐density (*N* = number of gas molecules per unit volume), and is thus related to the gas pressure (*p*) and temperature (*T*). For this reason, more useful for inter‐laboratory comparisons is the reduced mobility (*K*_0_), which is calculated from *K* by applying the following correction factor:

(2) $$K_{0}=K\cdot \frac{N}{N_{0}}=K\cdot \frac{p}{p_{0}}\cdot \frac{T_{0}}{T}$$

*N*_0_ is Loschmidt's number. The reference state commonly used in the IM community is a standard pressure *p*_0_ = 1 atm (101 325 Pa) and standard temperature *T*_0_ = 273.15 K. However, since 1982, the IUPAC recommends that the standard temperature and pressure (STP) should be used instead: *T*_0_ = 0°C (273.15 K) and *p*_0_ = 1 bar (100 000 Pa) (IUPAC, 1997). This illustrates that specifying the constants and conventions used is also crucial for data reuse: failing to specify which value of *p*_0_ was used introduces an uncertainty of 1.3 % in the definition of *K*_0_.

It is important to note that *K*_0_ still depends on the temperature *T*, on *E*/*N*, and on the nature of the buffer gas:

1. The temperature influences the structure (or conformational ensemble) of the ion (Mao et al., 1999; Kinnear, Hartings, & Jarrold, 2001; Gidden & Bowers, 2002; Kinnear, Hartings, & Jarrold, 2002). In polarizable gases (CO_2_, SF_6_, air, etc.), the temperature influences the formation of transient clusters and hence the reduced mobility as well (Karpas, Berant, & Shahal, 1989).
2. The reduced ion mobility also depends on the ratio *E*/*N*:
  (3) $$K_{0}\left(\frac{E}{N}\right)=K_{0}\left(0\right)\left[1+a_{2}{\left(\frac{E}{N}\right)}^{2}+a_{4}{\left(\frac{E}{N}\right)}^{4}+\cdots \right]$$
  Linear methods in the low‐field limit assume that *E*/*N* is low enough so that *K*_0_ = *K*_0_(0), whereas nonlinear methods exploit the *E*/*N* dependence (see section III). It is presently unclear how low *E*/*N* must be for the linear regime to remain valid (Hauck et al., 2017a), but because for future data exploitation it is better to err on the side of providing too many details than too few, it is recommended to report an estimate of *E*/*N* (or more conveniently, *E*, *p* and *T*) at which the experiments were carried out, even if it is thought to be in the low‐field limit.
3. Finally, the nature of the buffer gas influences the resisting force because it influences the nature of the ion‐gas interaction (gas size and polarizability) and the elasticity of collisions (long‐range interactions vs. hard‐spheres collisions) (Wyttenbach et al., 1997).

In summary, the temperature, the gas composition, and *E*/*N* should be part of the definition of the measurand in ion mobility. In practice, the level of detail and precision to which the measurand should be defined depends on whether the definition of the measurand is the limiting factor, compared to measurement uncertainty. For example, Hill's group recently reported on an instrument capable of measuring *K*_0_ values with 0.1% precision (Hauck et al., 2017b), which revealed the necessity of defining the measurand more precisely in terms of temperature range, gas composition (in particular, water humidity) and *E*/*N* range (Hauck et al., 2018). The gas composition was the major contributor to changes in *K*_0_. For many applications, it will probably be sufficient to state the gas identity and purity, a temperature range spanning no more than a couple of Kelvin (achievable in a temperature‐controlled laboratory), and to assume that *K*_0_ is independent of *E*/*N*. Providing the actual ranges and values will further help the reader to assess and interpret the results in light of the above discussion.

### D. Collision Cross Section (*CCS* or Ω)

To calculate the experimental *CCS* value from the mobility *K* or reduced mobility *K*_0_, most procedures use the mathematical function provided by the fundamental low‐field ion mobility equation (Revercomb & Mason, 1975):

(4) $$CCS=\frac{3}{16}\sqrt{\frac{2\pi }{\mu k_{B}T}}\frac{ze}{NK}=\frac{3}{16}\sqrt{\frac{2\pi }{\mu k_{B}T}}\frac{ze}{N_{0}\left(\frac{p}{p_{0}}\cdot \frac{T_{0}}{T}\right)K}=\frac{3}{16}\sqrt{\frac{2\pi }{\mu k_{B}T}}\frac{ze}{N_{0}K_{0}}$$

*μ* is the reduced mass of the ion‐gas pair: *μ* = *m_i_m_g_*/(*m_i_* + *m_g_*), where *m_i_* is the mass of the ion and *m_g_* is the mass of the gas, and *z* is the ion absolute charge. The relationship between *CCS* and *K*_0_ depends on the temperature. To give an idea of order of magnitude of the combined effect of the ion‐neutral interactions plus the temperature dependence of Equation (4), we can examine the trajectory model calculations for C_60_ (no possible conformational change) (Young & Bleiholder, 2017). The simulations give a relative CCS change of 0.13% per Kelvin around 300 K for C_60_^+^ in nitrogen (0.07% in helium).

Because Equation (4) is derived from the equilibrium of forward acceleration of the ion in the electric field and the opposing resisting force due to collisions with the buffer gas (momentum transfer to the buffer gas), *CCS* represents a *momentum transfer cross section* Ω. Historically, the term *collision cross section* was used in the context of a hard sphere collision model (Millikan, 1923) and is strictly speaking not entirely appropriate for the mobility‐derived cross section. The cross section based on the size of colliding spheres can differ from Ω by a factor of up to 1.4, or be identical to Ω in other cases, depending on the nature of the collisions (Wyttenbach, Bleiholder, & Bowers, 2013). Nevertheless, *CCS* is now routinely used to describe the quantity obtained from Equation (4). Thus, although the term collision cross section (symbol: *CCS*) is accepted, it should, however, be understood that it reflects a momentum transfer cross section (symbol: Ω). Both symbols are acceptable. We will use *CCS* in the remainder of the text.

It is important to note that the primary output of the measurement is the mobility (*K*), that the experimental *CCS* is a quantity derived from *K* via a mathematical model. In other words, *ion mobility measures mobilities, not surfaces*. A mathematical model such as Equation (4) is a compromise based on our best understanding of the physics of the phenomenon, and on some simplifications. Approximations underlying Equation (4) include that the electric field is zero (i.e., the kinetic energy imparted by the field is negligible compared to the thermal kinetic energy), which is fundamentally not the case in a real IM experiment (Siems, Viehland, & Hill, 2016). Thus, the use of Equation (4)—or any mathematical model—in the data treatment procedure constitutes also a possible source of bias. At the current state‐of‐the‐art we do not yet know the sign and magnitude of the correction factor that could improve the accuracy of the estimated value, although progress is being made in this respect (Siems, Viehland, & Hill, 2016). In principle both signs may be encountered, as the *K* values increase with increasing *E*/*N* for some ion/gas combinations (type A or B in FAIMS, see section V.D.3) and decrease for others (type C) (Barnett et al., 2000; Shvartsburg, 2009). We recommend that every equation and mathematical model used in the data treatment procedure should be stated clearly. If a mathematical model other than Equation (4) is used, it must be fully described.

Another consequence of the above is that, while ion mobility (*K* or *K*_0_) values can be traced back to the SI (its SI base unit is the A s^2^  kg^−1^, see Table 2) because the measurement consists in determining *v*_d_ and *E*, the CCS values cannot. The SI base unit of the CCS is the square meter, but ion mobility experiments do not measure areas. The CCS is a derived quantity. This discussion supports the following recommendations. (i) One should ideally report both *K* (or *K*_0_) and *CCS* values in databases, not *CCS* values alone. Another advantage is that, should new knowledge become available and alternative equations to Equation (4) become recommended, a re‐evaluation of *CCS* values from mobility values would be facilitated. (ii) The calibration of the instruments should be based on mobility values, not on CCS values. If only CCS values are available, they should first be converted to mobilities with an appropriate mathematical model, for example, based on Equation (4) if possible.

**Table 2:**
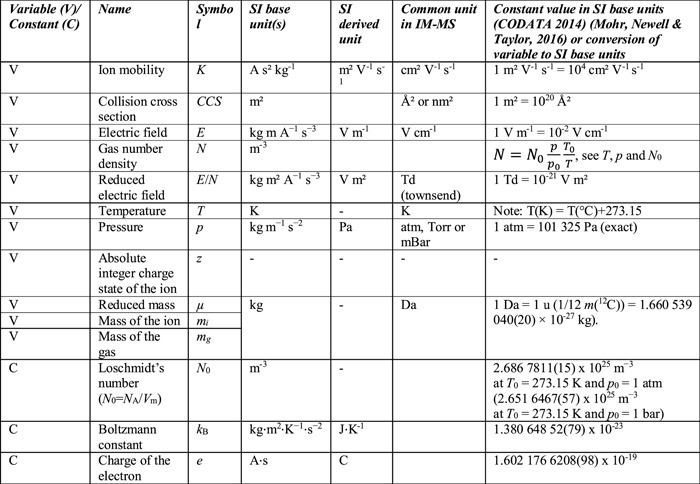
Variable and constants in SI units, and in units commonly used in IM‐MS

### E. Notation for Mobilities and Collision Cross Sections

As discussed above, for a given ion structure, *K*_0_ (and thus the *CCS* derived from it) depends on the gas composition, on the temperature, and on *E*/*N*. Besides a full description in the materials and methods section, it is desirable to include some metadata about gas, temperature and *E*/*N* in the *K*_0_ and CCS notation themselves. It is also desirable to have an explicit notation for values obtained by the primary method, versus secondary or empirical methods. As discussed elsewhere (Gabelica & Marklund, 2018), the range of fields used in so‐called linear methods can differ enough so that effective temperatures higher than the gas temperature may be reached in TWIMS, TIMS, or in DTIMS at high fields. Further, *K*_0_ and *CCS* depend on the population distribution of ion structures sampled in the experiment, which can be different for different experimental setups. Thus, although the principle of the measurement technique should not have an effect on the CCS *per se* (see also section III.C.2), specifying the instrument used currently makes sense. In a compendium of collision cross section values, the McLean group (May, Morris, & McLean, 2017) recommended using the nomenclature introduced by the Barran group (Pacholarz & Barran, 2015). This includes the method type as a superscript and the drift gas, or drift gas equivalent for calibrated values, as subscript, giving notations such as ^DT^ *CCS*_N2_ and ^TW^CCS_N2_, for CCS values obtained using a drift tube instrument with nitrogen gas and a traveling wave instrument using nitrogen gas, respectively.

Furthermore, we recommend an explicit notation to distinguish primary values (obtained without calibration) and secondary values (obtained following calibration). This is important because on the one hand not all DT measurements have the qualities of a primary method, and on the other hand attempts to determine CCS values directly (without calibration) from TWIM data are emerging (Mortensen, Susa, & Williams, 2017; Richardson, Langridge, & Giles, 2018). One way to specify this would be to include the notation “1ry” (primary) explicitly when appropriate, for example ^DT,1ry^ *CCS*_He_. This would single out the primary values, which are of special interest for calibrating secondary methods. For secondary methods, if gas 1 is the (predominant) drift gas in which the measurement was made, and gas 2 is the drift gas in which the calibrant values were obtained, we suggest using the notation: ^TW^ *CCS*_gas1→gas2_. It should also be noted that mobility values for gas mixtures can be determined and related to the partial pressures of the individual gases comprising the mixture through Blanc's Law (Revercomb & Mason, 1975).

For DT data, the buffer gas temperature is at present not included in the notation. Most measurements are performed in ambient conditions, that is, usually between 293 and 300 K. If temperature‐variable instruments (von Helden, Wyttenbach, & Bowers, 1995; Dugourd et al., 1997; Mao et al., 1999; Wyttenbach, Kemper, & Bowers, 2001; May & Russell, 2011; Ujma et al., 2016) become more widespread, or if measurement uncertainty becomes small enough that the exact temperature matters and the notion of “ambient conditions” is no longer sufficient, then introducing the temperature in the notation will become useful. This could come as an index next to the gas, for example: ^DT,1ry^ *CCS*_He,298_.

### F. Recommended Units and Values of Constants

Table 2 gives the units of each term used in Equations (1), (2), (3), (4), in SI base units, in derived SI units, and in commonly used non‐SI units, as well as the values of the constants used in Equations (1), (2), (3), (4) according to the 2014 CODATA (Mohr, Newell, & Taylor, 2016). Although these are not SI units, it is customary to report *K*_0_ values in cm^2^ V^−1^ s^−1^ and *CCS* values in Å^2^ or nm^2^. For practical reasons, using these units will be continued. Every report should specify the unit associated with each value.

Some literature values have been determined using older values of constants, or using fewer significant digits. Note also that a revision of the international system of units (SI) will take place in 2018 (BIPM, 2018) (formal decision in November 2018, implementation date May 20, 2019). It is thus recommended that the values of each constant used in the procedure are specified exactly as used (including *p*_0_ and thus *N*_0_, see section II.C) to ensure data reusability and eventual conversion. If a software or spreadsheet is used, these values may be hidden, and may change according to software and/or version. Ideally the values must be known and included in the reporting, but if these values are inaccessible, the software name and version should be specified.

## III. CLASSIFICATION OF IM‐MS METHODS

**Figure 1:**
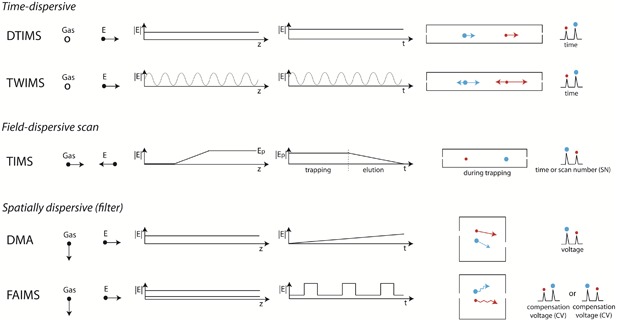
Classification of IM measurement principles discussed herein. From left to right: acronym (DTIMS = drift tube IMS; TWIMS = traveling wave IMS; TIMS = trapped IMS; DMA = differential mobility analyzers; FAIMS = field‐asymmetric waveform IMS), gas direction, field direction, electric field profile along the device (for DMA and FAIMS, assuming planar gaps; for FAIMS, the two lines indicate the high field and the low field), time profile of the electric field, schematic ion movement in the device, and typical readout.

Workflows differ according to the *principle of measurement*, linked to the type of instrument hardware (see Fig. 1), and according to the *objective of the measurement*.

### A. Principles of Measurement

#### 1. Linear Methods

In linear methods, *K*_0_ is *assumed* to be independent of the electric field. The measured property (usually the transit time) is thus *assumed* to be (inversely) proportional to the field. Linear methods include:

– DTIMS (drift tube ion mobility spectrometry), in which ions are directed through a stationary gas by a constant and homogeneous electric field *E* (Eiceman & Karpas, 2005). Radio‐frequency fields can be superimposed on the drift field to confine ions to the axis of separation, as in radio‐frequency confining drift cells (Allen & Bush, 2016; Allen et al., 2016). The *E*/*N* values for drift tube typically vary from 1 to 15 Td (1 Td = 10^–21^ V m^2^).
– TWIMS (traveling wave ion mobility spectrometry), in which ions are directed through a stationary gas by a sequence of symmetric potential waves continually propagating through the drift region (Giles et al., 2004). The ions are subjected to varying *E*/*N*, with peak values reaching 50–160 Td (Giles et al., 2010; Gabelica & Marklund, 2018). Structures for lossless ion manipulation (SLIM) can be used to implement traveling waves (Tolmachev et al., 2014) and thus perform TWIMS separations (Deng et al., 2017; Ibrahim et al., 2017).
– TIMS (trapped ion mobility spectrometry), in which ions are held in place against a moving gas with an electric field (Fernandez‐Lima et al., 2011). Multiple isomers/conformers are trapped simultaneously at different *E* values resulting from a voltage gradient applied across the IMS tunnel region. The typical *E*/*N* is 45–85 Td (Gabelica & Marklund, 2018). Ions are then released by decreasing the electric field in steps. Each isomer/conformer eluting from the TIMS cell can be described by a characteristic elution voltage (*V*_e_), which is connected to the 1/*K_0_* value (see section V.E.4).
– DMA (differential mobility analyzers), in which ions are separated spatially based upon electrophoretic migration in one direction (at mobility dependent speeds) and fluid flow driven migration in an orthogonal direction (with all ions at the same speed) (Reischl, 1991). Only ions of a prescribed mobility traverse from the inlet to the outlet of a DMA. DMA can be operated in the low‐field limit (typical *E*/*N* below 20 Td (Hogan & Fernandez de la Mora, 2009)).

#### 2. Nonlinear Methods

*Differential Ion Mobility Spectrometry* (DIMS) exploits the fact that the mobility (*K*) of any ion in any gas depends on the electric field intensity (*E*) to identify chemical species and separate their mixtures based on the *difference* between *K* values at two or more *E* levels (or ranges thereof) (Guevremont, 2004). It is a nonlinear IMS method in the sense that the measured perturbation in media (dynamics of the ion swarm) is not linear with respect to the magnitude of perturbing force (applied field) (Shvartsburg, 2009).

Except for the cases of electric dipole alignment, the *K* values depend on *E via* normalized mobility (*E*/*N*), see Equation (3). As varying *E* is a lot more practical, precise, and rapid than varying *N*, all present systems involve varying *E*. Experimentally, *E* is temporally varied in a periodic *asymmetric* waveform (i.e., the segments of opposite polarity are not mirror images). Hence the technique is alternatively called *Field Asymmetric Waveform Ion Mobility Spectrometry* (FAIMS). The shape of the enclosure (gap) where the field is established and separation is performed affects the field homogeneity, which is critical to DIMS system performance (Guevremont & Purves, 1999). For historical reasons, the systems with planar and curved gaps have preferentially been called *Differential IMS* and *FAIMS*, respectively. That distinction has no basis outside of dated historical context and is discouraged. To unify the associated literature and simplify its search, we suggest mentioning both *Differential IMS* and *FAIMS* in the abstract and/or introduction of publications as an interim measure until one term is settled upon. The term *Differential Mobility Spectrometry* (DMS) is often encountered too, primarily by users of the commercial SCIEX SelexION® (Campbell, Le Blanc, & Kibbey, 2015). A further term “Ion Mobility Increment Spectrometry” (IMIS) is common in the Russian‐origin literature (Buryakov, 2004). Thus, the four acronyms (DIMS, DMS, FAIMS and IMIS) designate the same principle of measurement. Below we will use “FAIMS” for conciseness.

### B. Types of Output: IM‐MS Data, *K*_0_ Values, and *CCS* Values

**Figure 2:**
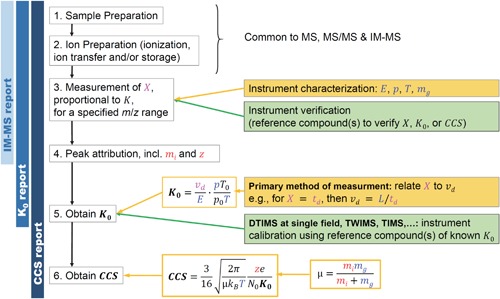
Typical workflow of IM‐MS experiments: steps 1–3 involve experiments, steps 4–5 involve data processing. Reporting IM‐MS data involves steps 1–4, reporting *K*_0_ values involves steps 1–5, reporting *CCS* values involves steps 1–6. Key equations defining the reduced mobility *K*_0_ and the *CCS* are given, with the input variables in each equation shown in red (ion‐related input variables), blue (variables related to the experimental setup) and magenta (variables deduced from the measurement).

A typical ion mobility mass spectrometry experiment involves up to six steps (Fig. 2): (1) sample preparation; (2) ion preparation (i.e., ionization, transfer, and storage); (3) IM‐MS measurement; (4) peak attribution; (5) mobility (*K* or *K*_0_) determination; and (6) CCS determination. Steps 1–3 involve carrying out the experiments and steps 4–6 involve data processing.

Steps 1, 2, and 4 are common to all mass spectrometry experiments. Their reporting guidelines are thus largely inspired by similar guidelines for reporting proteomics experiments or mass spectrometry data in proteomics (Taylor et al., 2007) and glycomics (Struwe et al., 2016). Step 3 is common to all ion mobility principles outlined above. For many applications which do not need mobility or CCS values, for example, in separation sciences or discussion of *changes* in ion structure (rather than absolute values), reporting IM data consists of steps 1–4. We thus include specific guidelines for reporting either linear or nonlinear (differential) mobility experiments, for example in the form of arrival time distributions. The determination of mobility values (step 5) can be done at any electric field, but only *via* linear (not differential) methods. The CCS values (step 6) can only be determined from experiments operating—or assumed to be operating—within the low‐field limit.

We thus distinguish three types of reports, each having different scopes and constraints: reporting IM‐MS data (steps 1–4), reporting *K*_0_ values (steps 1–5) and reporting collision cross section values (steps 1–6).

### C. *K*_0_ and *CCS* Determination: Methods of Measurement

#### 1. Primary Versus Secondary Methods

A primary method of measurement is defined (BIPM, 1995; Quinn, 1997; Milton & Quinn, 2001; Taylor, Kipphardt, & De Bièvre, 2001) as “a method having the highest metrological qualities, whose operation can be completely described and understood, for which a complete uncertainty statement can be written in terms of the SI, and whose results are, therefore, accepted without reference to a standard of the quantity being measured” (BIPM, 1995). For a complete uncertainty statement, the operation of the method should be represented by a measurement equation. The determination of *K*_0_ by DTIMS satisfies this definition with the least uncertainties: the constant field makes it straightforward to calculate *E* from Δ*V*/*L* (V m^−1^) and *v*_d_ from *t*_d_/*L* (s m^−1^) to obtain *K*_0_ (m^2^ V^−1^ s^−1^). Only few instruments are conceived to measure drift times *t*_d_ directly (Hauck et al., 2018). However, most instruments measure the arrival time (*t*_A_) at a location further away from the end of the drift region. Additional mathematical operations are required to obtain *t*_d_ from the measured arrival time *t*_A_, but these are also traceable (see an example in section V.E.2). Thus, *K*_0_ determined using DTIMS from first principles are today considered as primary standards. The *CCS* is then *derived* from *K*_0_ with a mathematical model such as Equation (4), but the CCS is not what is primarily *measured* by DTIMS.

Secondary methods use calibration against some primary standards or primary methods. It is important to underline that not all DTIMS measurement methods are primary methods. Working with a reference compound in DTIMS is preferable if the accuracies of *E*, *L*, *T* and *p* needed to implement the primary method are insufficient, while high‐quality reference values are available. The DTIMS methods include (I) a direct measurement of drift time (the method is more common in portable IM instruments with ^63^Ni radioactive ionization sources), (II) multi‐field measurement of arrival times to deduce the drift time from a linear regression and determination of the pressure from an instrument reading, (III) multi‐field measurement of arrival times and determination of the pressure using at least one reference compound, or (IV) single‐field measurement of arrival time, which is then converted to *K*_0_ with a calibration function *f* determined using several reference compounds of known *K*_0_ (or, more generally, *K*_0_, mass and charge). Thus, DTIMS methods (I) and (II) are primary methods, whereas DTIMS methods (III) and (IV) require reference compounds.

For TWIMS and TIMS, in practice the *K*_0_ and CCS values are determined *via* an instrumental calibration function *f* determined using reference compounds. With TIMS (Bleiholder, 2016) and TWIMS (Giles et al., 2010; Mortensen et al., 2017; Mortensen, Susa, & Williams, 2017; Richardson, Langridge, & Giles, 2018), obtaining *K*_0_ without calibration may be achievable in principle, but the uncertainty associated with such approaches in development has not yet been fully evaluated. In DMA measurements, precise knowledge of the applied voltage, DMA dimensions, and fluid flow rates into the device can yield *K*_0_ values without the need for calibration. DMAs can thus be used as a primary method of measurement; this approach has been shown accurate enough to examine the sizes of NIST certified polystryene latex size standard particles (Mullholland et al., 2006). For extremely large spherical ions, the CCS can be linked to the diameter. However, higher resolving power DMAs utilize high sheath flows (hundreds of liters per minute), hence in many instances reference compounds are used in DMA calibration (Ude & Fernández de la Mora, 2005), particularly for DMAs interfaced with mass spectrometers.

To summarize, in Figure 2, the gold color indicates the workflow allowing one to estimate *K*_0_ and *CCS* values without using any reference compound (primary method of measurement). This workflow is currently routine in DTIMS only. The green color indicates the workflows using reference compounds (compounds of known *K*_0_ measured across multiple instruments and laboratories) to verify and/or calibrate the instrument. The calibration procedure differs for DTIMS, TWIMS or TIMS in the sense that the function *f* differs, but common principles can be applied.

#### 2. Are Some Secondary Methods Empirical Methods?

In empirical approaches, the measurand is defined by the method itself (EURACHEM/CITAC, 2012). The measurand in ion mobility is defined by the ion structure, the gas, the temperature, and *E*/*N*. For a particular secondary method, if it is possible to place oneself in the exact same conditions of ion structure, gas, temperature and *E*/*N* as the primary method, then it is not an empirical method. However, this is not always possible. If we take the example of TWIMS, although efforts have been made to construct RF‐confining drift cells and fit them in a SYNAPT G1 (Bush et al., 2010) and G2 (Allen et al., 2016) to reproduce ion structures, gas and temperature conditions, TWIMS operates with inherently different *E*/*N* conditions than the RF‐confining drift cell. Thus, the TWIMS measurement method is empirical and may thus produce method‐dependent results (the calibrant choice being part of the method) even after partial correction through calibration. The accuracy of calibrated mobilities and dependence on the traveling‐wave method have been characterized for peptides (Bush, Campuzano, & Robinson, 2012) and protein complexes (Zhong, Hyung, & Ruotolo, 2011).

## IV. REFERENCE MATERIALS FOR ION MOBILITY

### A. Absence of Primary Standards for Ion Mobility

Primary measurement methods can serve to determine the values associated with primary standards (BIPM, 1995; Quinn, 1997), defined as “a standard that is designated or widely acknowledged as having the highest metrological qualities and whose value is accepted without reference to other standards of the same quantity” (BIPM, 1995). Primary standards serve to prepare certified reference materials (CRM). Then, according to recommended practice (EURACHEM/CITAC, 2012), any additional bias associated with another method should be estimated by comparing the results with those of a reference method or by analyzing an independent CRM as quality control. Unfortunately, there is not presently *consensus* for a primary standard compound and thus no reference material is currently available.

The initiation of a certification study on materials proposed as primary standards and on their associated mobility values is therefore urgent for our field. Meanwhile, we shall provide practical advice and recommendations on using the data currently available from the literature.

### B. Uncertainty Associated With Published Values Determined by the Primary (DTIMS) Method

Multiple papers have reported CCS values determined with the primary method (reviewed for IM (Kaur‐Atwal et al., 2009) and IM‐MS (May, Morris, & McLean, 2017)), which were subsequently used to calibrate or to validate other methods. The problem is that most published databases do not include an evaluation of the uncertainty, or if they did, not according to the current international guidelines (JCGM, 2008). The combined standard uncertainty associated with the primary standards is important to subsequently evaluate the combined uncertainty obtained by secondary methods. Without knowing the uncertainty associated with the calibrant values, it is impossible to evaluate the uncertainty of the output values.

#### 1. Standard Deviation of Replicated Experiments

When provided, the standard deviation of replicated experiments (*u*) indicates the dispersion of the experimental data (precision) under repeatability conditions, which contributes to—but is not equal to—the combined standard uncertainty. The number of independent technical replicates (*n*) is important to determine the coverage factor *k*_p_ required to calculate an expanded uncertainty (*U*_p_ = *k*_p_.*u*) associated with a certain level of confidence. For example, for a 95% level of confidence, *k*_p_ = 1.96 only if *n* = ∞. If *n* = 6, *k*_p_ = 2.57, if *n* = 3, *k*_p_ = 4.30 and if *n* = 2, *k*_p_ = 12.71 (JCGM, 2008).

#### 2. Combined Standard Uncertainty

A few studies provide a more detailed assessment of the combined standard uncertainty (Crawford et al., 2012; Stow et al., 2017; Hauck et al., 2018), based on proper propagation of the standard uncertainties on *t*, *E* (Δ*V*/*L)*, *p* and *T*. The work referenced above also devoted particular care to characterize *V*, *L*, *p* and *T* and their uncertainties with the appropriate technologies and measurement methods, not just by using instrument settings or read backs and ballpark estimates of the associated errors.

#### 3. How Do Values Obtained From Different Laboratories Currently Compare?

However, these approaches do not include an analysis of bias. The most appropriate way to assess trueness is to carry out a round robin test (an interlaboratory test performed independently—i.e., without knowledge of any expected value before submitting the results—several times). After discussion to define *consensus* values, the compounds could then be used to produce reference materials, having fixed reference CCS values accepted by the entire IMS community (“IUPAC compliant”). However, such interlaboratory study has not been reported to date.

**Figure 3:**
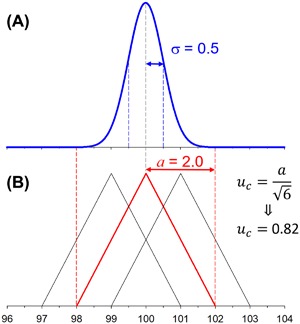
(**A**) Gauss‐Laplace distribution of typical results obtained from different laboratories with different DTIMS designs, for a hypothetical collision cross section of 100 Å^2^. The standard deviation σ is 0.5%, meaning that a span of 2% (±2s.d.) contains ∼95% of the values and that a span of 3% (±3s.d.) contains ∼99.5% of the values. (**B**) Graphical justification for the estimation of the standard uncertainty that should be associated to a value measured on one single DTIMS instrument.

Meanwhile, information can be gleaned by comparing values obtained by different groups. For the present paper, the groups of Bowers, Clemmer and Bush shared their experience in comparing CCS values obtained independently (Dugourd et al., 1997; Counterman et al., 1998; Bush et al., 2010; Dilger et al., 2012; Allen et al., 2016). The values found using different tube designs had a typical relative span of 2%, with 3% in a few cases. The *consensus* is thus that primary values measured using different DTIMS platforms have a distribution characterized by a relative standard deviation (s.d.) of 0.5% (see Fig. 3A). Note that the labs may have operated at slightly different ambient temperatures. Importantly, this distribution held true only *provided that the same ion structures were measured*.

In practice, in individual publications, values are reported for a *single* instrument platform. In such case, the combined uncertainty can be evaluated by a type B approach (JCGM, 2008). Figure 3B shows that assuming triangular distributions with a 2% margin on each side is reasonable. With these assumptions, one can assign to published values a combined standard relative uncertainty of ≈0.8% (expanded uncertainty with a 95% level of confidence: ≈1.6%). This is the best estimate of the level to which measurements across instrument platforms with different designs and in “ambient” conditions can compare today. Reducing this contribution to uncertainty will require the community to agree on at least one reference value to which all measurements could be traced.

### C. Recommendations for Choosing Future Reference Materials

Reference compounds for *calibration* should be primary standards that are the least sensitive to sample manipulation (chemically stable upon long‐term storage and dilution in different solvents), to ionization (the same ions should be produced independently of the ionization method, solvent used, flow rate, etc.). to ion‐molecule reactions in the source or transfer regions, and to ion activation. In other words, ideally all users should measure the same ion structures, independently on what instrument types and settings they choose. Further, the reference compounds should ideally be robust in terms of variations of quantities that affect the definition of the measurand (temperature, moisture, and field) (Kaur‐Atwal et al., 2009; Hauck et al., 2018), or have characterized dependencies that can be used to determine correction factors and their associated uncertainty.

In MALDI‐IMS, a commonly used reference compound was C_60_^+^ (Von Helden et al., 1993; Dugourd et al., 1997), but unfortunately this ion is not particularly suitable for analysis in common ESI solvents. For ESI, other calibrants were thus used (Wyttenbach, Von Helden, & Bowers, 1996; Hoaglund et al., 1997; Shelimov et al., 1997; Valentine et al., 1997; Valentine, Counterman, & Clemmer, 1997; Counterman et al., 1998; Hoaglund et al., 1998; Wyttenbach, Bushnell, & Bowers, 1998; Henderson et al., 1999,1999; Valentine, Counterman & Clemmer, 1999; Fenn et al., 2009; Bush et al., 2010; Bush, Campuzano, & Robinson, 2012; Campuzano et al., 2012; Salbo et al., 2012; Forsythe et al., 2015; May, Morris, & McLean, 2017). The hexakis‐fluoropropoxyphospahzines (Agilent Tunemix calibration standard (Stow et al., 2017)) and polyalanine (Allen et al., 2016) satisfy the criteria and are compatible with ESI. Several other synthetic polymers (Duez et al., 2017; Haler et al., 2017, 2018) or supramolecular assemblies (Hupin et al., 2018) have recently been proposed as alternative calibrants. Finding new reference materials is currently a very active area of research, and it is expected that a few more years of testing will be necessary to reach *consensus* on the species and their mobilities. It is also likely that different chemical classes of reference materials will have to be developed, depending on the analyte (e.g., to ensure interpolation in the *K*_0_ domain (Shvartsburg & Smith, 2008), and for TWIMS, to have similar *K*_0_ = *f*(*m*/*z*) dependencies (Richardson, Langridge, & Giles, 2018)).

Besides a set of compounds for calibration, an independent *quality control* should be used. Regularly monitoring the value of its estimated *CCS* within a specified uncertainty window, with the same experimental settings as for the calibration and *K*_0_ or *CCS* determination, will unveil systematic deviations with time, and indicate when recalibration is necessary. A QC reference compound is also used to estimate the trueness of the method when necessary (when absolute values are the goal).

In addition, the community would also benefit from having reference compounds for *instrument performance evaluation*, that is, compounds which would be sensitive to some particular aspects of the measurement, such as the pre‐IM activation (e.g., native ubiquitin^7+^ is frequently used to gauge instrument softness (Wyttenbach & Bowers, 2011; Chen & Russell, 2015; Gabelica, Livet, & Rosu, 2018)), post‐IM activation (a fragile compound), sensitivity to gas purity (in particular, sensitivity to moisture), temperature, or *E*/*N*. Periodic monitoring of one or more reference compounds may be used to correct small deviations in measured drift times (or equivalent quantities) during long experiments (e.g., LC‐MS analyses), in a similar way as when correcting the mass bias using a lock‐mass strategy for TOFMS.

## V. REPORTING ION MOBILITY MEASUREMENTS

### A. Sample Preparation and Ion Preparation (Steps 1–2)

These steps influence the nature of the ion population that is subjected to measurement from a given analyte (see Section II.A). The different charge isomers (e.g., protonation isomers), the different adducts, the ions of different polarities and charge states, and the conformers introduced into the mobility cell must be considered as *different ions* produced from the *same analyte* in the sample, each potentially having different mobilities. It is thus normal that databases contain *CCS* and *K*_0_ values for different ions corresponding to a same analyte entry. If there is mass selection or significant discrimination in transmission (e.g., quadrupole cut‐off) of the ion population admitted into the IMS separation region, the *m*/*z* range must be indicated (this is different from the *m*/*z* range selected during data processing, i.e., step 4).

In turn, the nature and relative abundance of the adduct(s) or charge isomers can depend on the sample preparation and ionization/ion transfer conditions. Similarly, ion activation (i.e., internal energy) history prior to the mobility measurement can potentially influence the nature of the charge isomer or adduct, its conformation, or both because they are linked. This justifies the importance of reporting as many instrumental parameters as possible (source conditions, solvent and flow rates, voltages, pressures, temperatures, transit times, accumulation times and trapping times, bias voltages for ion injection in the IM) along with the exact instrument type and geometry.

### B. Experimental Parameters (Step 3) for Linear Methods

The measured output *X* may differ depending on the measurement principle, but in all linear methods, the output *X* is linked to the ion drift velocity *v*_d_. The following sections discuss the experimental parameters that must be described to proceed toward steps 5 and 6. The necessary information differs for the first principle determination and for calibrated measurements. For first principles, the minimum is to report all variables and constants involved in Equations (1), (2), (3), (4), that is, *E*, *T*, *p*, and gas composition which will define *m*_g_. For calibrated methods, the report should focus on the consistency of the conditions between the calibrants and analytes, and on providing all necessary information to reproduce the results.

#### 1. Electric Field

Because *E* = Δ*V*/*L*, the general principle is to report instrument design and voltages. For the instrument design, if a primary article has described the instrument in detail, a citation suffices. Voltages should be specified in every report for clarity.

##### Drift tube IMS

The electric field is linear and constant. One can find *E* from the voltage difference (Δ*V*) and the length *L* of the drift region, (*E* = Δ*V*/*L*). Bias may occur if the voltage difference is not *monitored during* the measurement. Other possible sources of bias are field inhomogeneity, difference between the geometric length of the drift tube and the effective length over which Δ*V* is applied (Hauck et al., 2017a) (this effective length may depend on Δ*V* and on the field in the optics entering and exiting the drift tube), and gas dynamics. To minimize additional errors, it is imperative that users, when measuring the high voltage found in many drift tube IMS systems, correct for the degree to which the high voltage probe alters the observed voltage.

##### Traveling wave IMS

For TWIMS, name the specific instrument model if commercial (G1, G2, G2‐S, etc., which will specify the wave shape), and give the entered “wave height” (in Volts) and “wave velocity” (in m/s). Descriptions of the typical wave shapes have been published for the first‐generation SYNAPT (Giles et al., 2004), and the SYNAPT G2/G2‐S/G2‐Si (Giles, Williams, & Campuzano, 2011) series. For home‐made instruments (e.g., SLIM‐based), specify the electrode dimensions, inter‐surface gap, and all wave attributes (shape, height(s), velocity) (Hamid et al., 2018).

##### Trapped IMS

In a TIMS analyzer, the axial electric field *E* varies both temporally and spatially (Fernandez‐Lima et al., 2011; Michelmann et al., 2015; Bleiholder, 2016). Spatially, the electric field strength increases over the length of the ion storage region up to a value *E*_0_ in the first half of the analyzer (“ramp”) whereas it remains constant at *E*_0_ in the second half of the analyzer (“plateau”). Temporally, this electric field profile is controlled by the rate *β* at which the plateau height *E*_0_ is decreased (“ramp rate”). Mathematically, the electric field strength at axial location *z* in the analyzer at time *t* is given by (Michelmann et al., 2015; Bleiholder, 2016):

(5) $$E\left(z,t\right)=\{\begin{array}{c} s\left(z\right)\left(E_{0}-\beta t\right);\forall z<z_{0}\\ E_{0}-\beta t;\forall z\geq z_{0} \end{array}$$

where *z*_0_ denotes the location of the center in the TIMS analyzer, *s*(*z*) is a function that describes how the electric field strength increases over the length of the first half of the analyzer, and *β* is the electric field scan rate. The function *s*(*z*) depends on the exact configuration of the resistor chain that connects the electrodes in the analyzer region. In many TIMS systems (Fernandez‐Lima et al., 2011; Michelmann et al., 2015; Bleiholder, 2016) and in the current tandem‐TIMS implementation, *s*(*z*) = *z*/*z*_0_ but *s*(*z*) can be nonlinear in other systems.

During normal operation, only the voltages at the entrance (*V*_ramp_) and exit (*V*_out_) of the TIMS tunnel section and their variation with time are changed by the user (the actual values are currently accessed in service mode). The most common form of operation is a linear variation of the *V*_ramp_ with the ramp time (*t*_ramp_). The report should include the *V*_ramp_ range, ramp time (*t*_ramp_), and pressures in the entrance and exit (P1 and P2) (Hernandez et al., 2014). In the case of nonlinear scan operation of the TIMS device, the *V*_ramp_(*t*) profile should be reported (Silveira et al., 2016a; Benigni et al., 2018).

##### Differential mobility analyzers

DMAs can be found in parallel plate (Hogan & Fernandez de la Mora, 2009), concentric cylinder (Fernandez de la Mora et al., 1998), and radial geometries (Zhang et al., 2007). Ion motion is driven electrostatically in one direction, while a clean gas sheath flow drives ion motion in an orthogonal or near orthogonal direction, such that only ions of a specific mobility traverse the instrument from inlet to outlet. DMA operates with similar instrument geometries as nonlinear (FAIMS) methods, and section V.C can be consulted for the geometric details to be reported. Irrespective of geometry, the mobility of the transmitted ions is proportional to the product of instrument geometry constant and the sheath flow rate, and inversely proportional to the voltage difference between the inlet and outlet electrodes. For IMS measurements the following items should be reported: (i) the DMA classification region dimensions (or the commercial model specified, along with the DMA geometry), (ii) the voltage range applied in measurement, and the scan duration or rate.

#### 2. Gas Nature and Pressure (or Gas Composition and Partial Pressures)

Gas compositions should be reported as volume fractions (see also V.C.2). For the pressure, the principle is to specify how, where, and when the pressure is measured. Theoretically it is the pressure *inside* the drift region *during* the measurement (or the pressure drop along the analyzer in TIMS). It is recommended to specify how and where the pressure was determined, the type of gauge used and the model (remember that gauge specifications are gas and pressure‐dependent; e.g., Pirani gauges are very gas‐dependent, whereas capacitance manometers are gas‐independent and should thus be preferred). Furthermore, different instruments may suffer from different biases or fluctuations related to gas flows, and for this reason the verification of an instrument with a reference compound is often carried out instead of—or in addition to—the pressure measurement. Leakage of gas or moisture from the source to the drift region is a main source of contamination, and in this context, reporting the source and ion transfer parameters is also relevant to the characterization of the gas composition in the drift region.

##### Drift tube IMS

The measurements are carried out in pure gases for CCS determinations, but *K*_0_ determinations are possible in gas mixtures. Note that ensuring the gas purity can be challenging depending on the instrument geometry and isolation of source and mobility regions. For example, if the tube must operate in 100% helium at sub‐ambient pressures, pressure differentials must be carefully controlled to avoid penetration of nitrogen or air from the source into the drift region. Furthermore, pressure homogeneity and absence of net gas flow is often assumed, but difficult to achieve in practice.

##### Traveling wave IMS

In Synapt G1 instruments, the TWIMS region contains only one gas, usually nitrogen (assuming that argon does not leak from the trap and transfer cells). In Synapt G2 and G2‐S instruments, however, a helium cell immediately precedes the nitrogen‐containing TWIMS region. As a consequence, the TWIMS region actually contains a mixture of helium and nitrogen. The report should specify how the pressure was regulated: either by fixing the gas flow rates (provide the flow rate settings in mL/min and the range of pressure readbacks from the He and N_2_ cells) or by varying the flow rates so as to have a narrow range of pressure readbacks (Morsa, Gabelica, & De Pauw, 2011; Morsa, Gabelica, & De Pauw, 2014) (provide the measured pressures, specify the gauges used, and provide the typical range of gas flow rates used to achieve these readings).

##### Trapped IMS

In TIMS, the buffer gas is forced to stream through the analyzer and exert a force that pushes the analyte ions toward the exit of the analyzer. The buffer gas velocity profile is nearly homogenous in the central region (typically, tens to hundreds of m/s) but decreases near the walls of the TIMS tunnel region due to friction (Hernandez et al., 2014). The resulting parabolic flow profile determines the trapping conditions during TIMS operation; that is, the basis for the mobility separation lies in the compensation of the drag force (proportional to the gas velocity *v*_g_) by the electric force (increasing from the entrance to the exit). The operator modulates the velocity of gas stream through the TIMS analyzer (corresponding to the volumetric flow rate of the gas divided by the cross section area of the TIMS analyzer) by setting a pressure difference between the entrance and the exit of the analyzer (P1 and P2, respectively, which should be reported). More details can be found in the literature (Hernandez et al., 2014; Michelmann et al., 2015; Silveira et al., 2016b).

##### Differential mobility analyzers

As DMAs require using a controlled, steady, laminar flow (often at atmospheric pressure), reporting on gas composition and flow conditions is essential. Specifically, the gas composition, flow rate, temperature, and relative humidity need to be specified as precisely as possible. DMA hardware bears resemblance to nonlinear designs (though the manner of operation is quite distinct), and the reader can consult sections V.C.1 to V.C.3. for more detailed guidelines.

#### 3. Temperature

This parameter is essential to ensure reproducible data and determine *K*_0_ and *CCS* from first principles. The report should specify how, where and when the temperature is measured. Theoretically one should determine the temperature of the gas *inside* the drift region *during* the measurement. Temperature measurements made outside and inside the drift region may not exactly reflect the actual temperature ions experience. The temperature is one of the most difficult parameters to control and measure, possibly a major source of bias and fluctuations. When using “ambient” conditions, the actual laboratory temperature should be reported (ideally, the laboratory should be thermostated).

On the SYNAPT (commercial TWIMS) instruments, the temperature is not measured, and since measurement of both calibrants and analytes will be affected, only differential effects will appear in the calibrated results. Furthermore, in TWIMS and TIMS, the ions may encounter collisions at a higher relative velocity than the corresponding thermal velocity. The extent to which these effects affect the results is not yet clear. Because it is preferable to err on the side caution, by reporting rather too many details than too few, all parameters influencing the ion‐gas relative velocity should be mentioned. In TIMS, this is directly dependent on the gas velocity *v*_gas_. In TWIMS, the relevant instrumental parameters are the wave height, wave speed, and gas pressures. The mass of the gas, of the ion, and the ion charge will also influence the effective temperature (Morsa, Gabelica, & De Pauw, 2011; Morsa, Gabelica, & De Pauw, 2014).

### C. Experimental Parameters (Step 3) for Nonlinear Methods

#### 1. Gap Dimensions

The FAIMS *gap* is between a pair of electrodes carrying the asymmetric waveform (Guevremont, 2004). Ions are filtered by motion perpendicular to the gap (along the field direction) while pulled through it by gas flow. The metrics of the *gap* are *width g* (the distance between opposite electrodes), *length L* (the shortest distance along the median traversed by ions from entrance to exit), and *span s* (the lateral dimension perpendicular to width and length), typically specified in mm or µm (*g*) and cm or mm (*L* and *s*) (Shvartsburg, 2009). With non‐planar gaps, the curvature is specified by *shape* (cylindrical or spherical) and *electrode diameters* (outer for internal electrode and inner for external electrode, typically in mm) (Guevremont & Purves, 2005). More complex gap shapes require further dimensions, for example, the length of cylindrical section and range of *g* in the hemispherical section of “dome” geometry (Tang et al., 2005).

#### 2. Buffer Gas Properties

##### Composition

The buffer gases often comprise two gases or a vapor doped into a gas (especially for higher FAIMS resolution, exploiting chemical interactions between these gases and ions or deviations from the Blanc law for gas mixtures in high electric fields). The composition should be specified in *volume fractions* (typically percentages for gases (Shvartsburg, Tang, & Smith, 2004) and ppm for the vapors (Krylova et al., 2003)). Specifying the partial vapor pressure is discouraged, as the associated fraction depends on the temperature and total pressure.

##### Pressure

Historically, FAIMS operated at ambient pressure, including with defined gas compositions (rather than ambient air). The separation parameters scale roughly as the inverse pressure squared (Shvartsburg, 2009), and large pressure variations that can be caused by difference in altitude above the sea level and weather conditions and/or laboratory setup have resulted in major discrepancies between the parameters measured in different settings or at different times. Indicating the *ambient pressure* (obtained using a barometer near the FAIMS device, usually in Torr) is advised for future work (Canterbury et al., 2008), unless the pressure is controlled (as in some recent studies (Nazarov et al., 2006; Shvartsburg et al., 2018)), in which case the *measured pressure in the gap* should be stated. The variation of pressure can to some extent be offset by using calibrations (below). Some commercial FAIMS offerings, such as those by *SCIEX* and *Heartland MS*, account for such environmental variations in ambient pressure.

##### Temperature

The *gas temperature* is crucial. Historically, FAIMS was operated at ambient temperature, and its variations contribute to the discrepancies between analyses made in different places or times (Krylov, Coy, & Nazarov, 2009). Less preferably, in arrangements where the temperature of the FAIMS cell is controlled by heating the supplied carrier gas, the temperature measured near the unit inlet may be reported (Robinson et al., 2008). The temperature should be ideally measured on electrode surfaces facing the gap (e.g., using a thermocouple with the waveform off), or in close proximity to the cell unit but not in direct contact with the electrode itself (i.e., no electrical contact or influence on the thermocouple by the waveform) (Schneider et al., 2010). Some systems employ a *thermal gradient* between electrodes to create ion focusing in the gap (Barnett et al., 2007), and in such case the temperature of each electrode should be specified.

#### 3. Gas Flow and Residence Time

The residence time in the gap *t* (or *filtering time*) is proportional to the width and inverse *volume flow rate* (*Q*_gap_, normally in L/min). However, the often used (Canterbury et al., 2008) *t* = *gLs*/*Q*_gap_ is only an approximation because of the gas flow gradient across the gap forced by boundary conditions on the electrodes (Shvartsburg, Tang, & Smith, 2005). As ions exiting the gap have traversed it near the median where the gas flow is faster than the average, the above is an upper limit and actual values are substantially lower. Accurately, there is a distribution of *t* due to the longitudinal diffusion and inequivalent paths through the gap traversing regions with different gas flow speeds.

In most FAIMS instrument designs, the measured gas flow to the device (*Q*) splits between those to the source (for ion desolvation) and the gap (*Q*_gap_), hence *Q*_gap_ is not accurately known, although it can be estimated based on the calculated conductance limits along the two paths (Shvartsburg et al., 2006). In other systems (sealed to a mass spectrometer) with gas flow drawn by MS vacuum suction, attaching the FAIMS device affects the conductance into the mass spectrometer and actual *Q* is below that for the underlying MS system. Some systems of this type also add (pull) a measurable gas flow to (from) the FAIMS/MS interface (Shvartsburg et al., 2009), which must be accounted for when calculating *Q*.

Hence we recommend reporting *Q* and (if desired) *Q*_gap_ and *t* estimated as above with proper clarifications, unless it was directly measured—e.g., using a shutter (Shvartsburg et al., 2018). In the latter case, the actual *t* (preferably with the distribution) should be stated.

#### 4. Asymmetric Waveform

As with any periodic function, the first key metric of FAIMS waveform is *frequency*, to be specified in Hz (kHz, MHz). The waveform is often synthesized from individual (in particular, two) harmonics, and its frequency should not be confused with those of individual harmonics (Shvartsburg, Tang, & Smith, 2004; Shvartsburg, 2009). The second key metric is the peak amplitude termed *Dispersion Voltage* (*DV*), expressed in V (kV). To avoid confusion, *DV* should not refer to the waveform itself, and the peak‐to‐peak amplitude (common in electrical engineering literature) should not be given; instead, the zero‐to‐peak amplitude of the waveform should be stated.

Unlike with symmetric (harmonic) waveforms, defining an asymmetric waveform also requires specifying the *profile*. In general, the profile must be specified numerically *via* a graphic or table (Shvartsburg et al., 2009). However, the waveforms resulting from superposition of harmonics can be described analytically in their terms. For example, the common bisinusoidal waveform comprises two harmonics with fixed 1:2 frequency ratio and can be specified *via* their amplitude ratio (commonly 2:1). A nominally rectangular profile can be defined in these terms (as “high‐to‐low ratio” of maximum absolute voltages in opposite polarities), but the rise and fall times must be given (Shvartsburg et al., 2018). The key metrics of waveform profile are its moments <*F*_n_> that control the FAIMS separation parameters and resolution. Their number is theoretically infinite, but most important are those for *n* = 2–7 and especially 2, 3, 5 (Shvartsburg, 2009). Moreover, an asymmetric waveform has a specific *polarity*, defined by the sign of *DV*. This polarity is critical to analyses in curved gaps and must be reported.

In summary, the waveform is defined by frequency, *DV* (including sign), and profile specified directly (analytically or numerically) and/or indirectly *via* the set of relevant moments. Both profile descriptions are advisable for redundancy and higher accuracy (given the infinite number of <*F*_n_> moments and inherent inaccuracy of numerical definitions).

#### 5. Compensation Voltage

While the waveform disperses ions along different trajectories inside the enclosure, those allowed to exit to the detector are selected by *Compensation Voltage* (*CV*), expressed in V (Guevremont, 2004). With FAIMS operated in the prevailing scanning mode, CV should be specified *via range* (initial and final values) and *scan rate* (V/min or V/s). With operation in the fixed‐ or stepped‐CV modes (parallel to SIM and MRM in mass spectrometry), each CV and *step duration* (in min or s) should be specified (Canterbury et al., 2008). With equal steps, one can state the *CV range* and *increment* instead of each CV (Tang et al., 2005).

As with *DV*, the *sign* of CV is critical. The physical sign of CV depends on whether it is superposed on the waveform on same electrode or applied to the other electrode with fields superposed in the gap (Shvartsburg et al., 2006). The two arrangements (adopted in different instruments) formally yield opposite CV signs for identical separations. To address this problem, we propose the sign convention where CV is assumed superposed on the waveform regardless of the actual implementation. This yields CV>0 for ions with *K* values decreasing at higher *E*/*N* and CV < 0 for those with *K* increasing at higher *E*/*N* (Kaszycki, Baird, & Shvartsburg, 2018; Shvartsburg et al., 2018).

#### 6. FAIMS Operating Modes

The combinations of positive and negative ion charges and two signs of *K*(*E*) or *K(E/N)* derivative results in four useful FAIMS modes as defined by (Purves et al., 1998): P1 (cation with positive derivative), P2 (cation with negative derivative), N1 (anion with positive derivative), N2 (anion with negative derivative). We recommend summarizing the operating mode in those terms, even if the information involved is separately stated. Those modes define the correct waveform polarity for ion focusing in curved gaps with inhomogeneous electric fields: positive for P1 and N2 and negative for P2 and N1. The opposite polarities cause ion defocusing and are thus not analytically useful (Purves et al., 1998). Hence the above indication of FAIMS mode essentially contains the information about waveform polarity in curved‐gap systems (that in planar‐gap systems with homogeneous fields is immaterial).

### D. Reporting IM‐MS Data (Step 4)

#### 1 *m*/*z* Range

We recommend reporting the extracted mass range [(*m/z*)_1_, (*m/z*)_2_] for each IMS or FAIMS feature during data processing, to clarify which isotopologues (relevant to smaller molecules) or adducts (relevant to large biologically relevant complexes) are averaged in.

In linear IMS, the isotopologue effects come from a reduced mass factor (which is removed when converting mobilities to CCS). Any validated difference in CCS (not reported thus far) would indicate other effects, primarily the isotopic impact on the ion geometry that could be structurally informative. The isotopic effects in FAIMS are now proven to be highly structurally specific (allowing robust isomer differentiation) and not substantially mass‐dependent. The specification of isotopic envelopes was not considered in IMS until very recently (Valentine & Clemmer, 2009), as all isotopologues were previously indistinguishable because of limited resolution. With the improvements in resolving power of both linear IMS and FAIMS, separation of isotopologues is now possible (Shvartsburg, Clemmer, & Smith, 2010; Kaszycki, Bowman, & Shvartsburg, 2016; Kirk, Raddatz, & Zimmermann, 2017) and necessitates defining the isotopic composition of species in question. By analogy to MS, we propose in linear IMS the *monoisotopic ion mobility* (raw or reduced) for the monoisotopic species, *average mobility* for the integral over full mobility distribution for all natural isotopologues, and *most probable mobility* for the most abundant isotopologue. In FAIMS, we would respectively have the monoisotopic, average, or most probable compensation field (raw or normalized).

#### 2. Ion Mobility Spectra

These data are the relevant output when ion mobility is exploited for separation sciences. The measurable *X* is usually an arrival time distribution (see Section II.2). ATDs should be presented as follows: the *y*‐axis is the ion signal (number of counts or relative intensities), and the *x*‐axis is the time (usually labeled *t*_A_ for “arrival time”). Don't label the axis “drift time” if what is measured is an “arrival time”. Alternate graphs are possible, for example by converting arrival times to drift times (time spent in the drift region, *t*_d_), to the voltage of elution (*V*_e_) in TIMS, or to compensation voltage (CV) in FAIMS.

For structural studies, ATDs are also important to reveal peak width, peak shape, and the time the ions spent in the separation region (which can influence the result in case isomerization can occur during the separation (Laszlo, Munger, & Bush, 2017; Poyer et al., 2017)). In the framework of structural studies, reporting collision cross section distributions (CCSD) becomes increasingly popular. An outline and discussion of the different procedures possible for drift tube ion mobility is provided by Marchand et al. (2017). It should also be noted that these procedures introduces additional uncertainty and biases depending on the data processing method (using the term “apparent CCSD” would convey this notion), and should not be used when ions of different charges coexist within the given *m*/*z* range. The principle is to report all information required for traceability, that is, to enable the user to convert back and forth between CCSD and the ATD.

Reports focused on *K*_0_ and *CCS* values should preferably include representative arrival time distributions as supporting information, for example to show the peak width, the peak shape, and absence/presence of distinguishable minor contributions. If a peak fitting software was used to distinguish contributions, all details should be given so that the reader could reproduce the data processing. Mass‐to‐charge/drift time heat maps with the intensity represented by a color code are useful for visualization, but should be accompanied by the extracted ATD and mass spectra to give a better sense of the relative abundances. In the case of *CCS* reports, the charge assignment for each mobility peak should be specified, and supporting evidence for this assignment should ideally be documented (for example, with an extracted mass spectrum showing the isotopic distribution).

#### 3. Specific Recommendations for Nonlinear Methods

##### Expressing voltages in terms of field strength

Reported FAIMS systems have gap widths varying by >100‐fold (from 0.035 to 5 mm). As a result, dramatically different DV and CV values can provide similar separations (Shvartsburg et al., 2009; Shvartsburg et al., 2018). DV and CV can be employed in the reports only provided that FAIMS cell dimensions are also clearly stated (such that *E*_D_ and *E*_C_ could be calculated, see below). However, to help compare the FAIMS spectra across systems with different *g*, we recommend converting DV and CV (via dividing by *g*) into *Dispersion Field* (*E*_D_) and *Compensation Field* (*E*_C_), typically in V/cm (Kaszycki, Baird, & Shvartsburg, 2018). These quantities carry the signs of DV and CV. One can further convert *E*_D_ and *E*_C_ into *normalized dispersion* and *compensation fields E*_D_/*N* and *E*_C_/*N* expressed in units of Townsend (Td) (Shvartsburg et al., 2018). These units may find greater use to compare results across pressures, owing to the emergence of FAIMS units capable of operation above or below 1 atm. However, proteins and perhaps other macromolecules with strong macrodipoles may be reversibly aligned by strong field in FAIMS, a process governed by *E* (rather than *E*/*N* as other high‐field effects underlying FAIMS) (Shvartsburg et al., 2009). Hence separations may be controlled by a combination of *E* and *E*/*N*, and reporting just *E*/*N* is insufficient in those situations. As an interim solution, we propose reporting *E*_D_ and *E*_C_ for analyses at ∼1 atm (that so far dominate FAIMS applications) and *E*_D_/*N* and *E*_C_/*N* for substantially different pressures.

##### Complete *E*_C_(*E*_D_) curves

Many FAIMS analyses employ a single *E*_D_ value, either the highest allowed by the hardware or the *E*_D_ value that provides optimal separation of ions balanced with concomitant signal attenuation. This generally maximizes resolution, but may limit the analytical information as (i) fragile species may not survive to the maximum *E*_D_ because of dissociation or isomerization upon field heating and (ii) certain species may be better resolved at lower *E*_D_ (Shvartsburg et al., 2012). Also, the full *E*_C_(*E*_D_) curve allows extracting the *α*(*E*) function (below) that most specifically identifies any ion (Guevremont et al., 2001). Hence, we recommend presenting in graphic or tabulated form the *E*_C_(*E*_D_) or *E*_C_/*N*(*E*_D_/*N*) curves over the whole experimental *E*_D_ range rather than single *E*_D_ values.

##### Extracting *a*(*E*) functions from FAIMS data

The ultimate way to catalog FAIMS data is *via* its *alpha‐function* as a function of *E* (or *E*/*N*):

(6) a=K(E)/K(0)

where *K*(0) is the zero‐field mobility (Guevremont et al., 2001).

This most universal representation allows comparing the results independently of the gap width, gas pressure, and, most importantly, waveform profile inherent in the *E*_C_(*E*_D_) curves. Extracting the *a*(*E*) functions requires deconvoluting *E*_C_(*E*_D_) curves for the known waveform profile (Schneider et al., 2016), which appears to produce unique results for practical profiles (Shvartsburg, 2009). The resulting *a*(*E*) or *a*(*E*/*N*) functions could be reported in graphic forms or as tables. While this deconvolution involves significant effort, it is recommended because it produces more transferable data for comparisons across systems and laboratories, especially with the proliferation of different FAIMS systems employing diverse waveform profiles.

##### Using FAIMS data to reconstruct absolute *K*(*E*) functions

No information on absolute mobility is obtainable from FAIMS data. However, they can be combined with linear IMS measurements providing *K*(0) to construct the absolute *K*(*E*) or *K*(*E*/*N*) curves. Then the error margins of the *K*(0) point translate into resulting curves and its source should be verified and reported.

##### Ion types

All ions have been phenomenologically grouped into *three types* by FAIMS separation properties (Levin et al., 2006a; Shvartsburg, 2009; Rorrer & Yost, 2011). These types are handy to concisely describe the ion behaviors and associated FAIMS regimes, because they can be readily visualized from the experimental data (Levin et al., 2006b; Rorrer & Yost, 2011). The *type* A and C ions were defined to have *E*_C_(*E*_D_) functions uniformly increasing and decreasing at higher absolute *E*_D_, respectively. For *type* B ions, *E*_C_ would first increase and then decrease with increasing absolute *E*_D_. These definitions were made with an early sign assumption for *E*_C_. With the present uniform sign convention, these definitions are amended to those listed in Table 3.

**Table 3:**
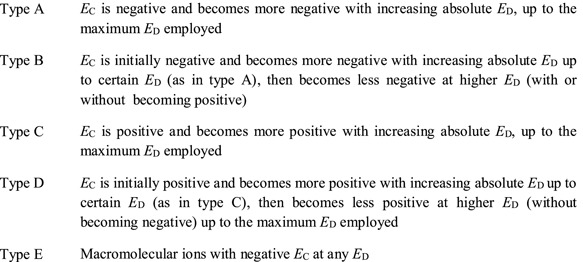
Definition of ion types in nonlinear methods

These definitions naturally expand to *E*_C_ and *E*_D_ expressed as *E*_C_/*N* and *E*_D_/*N*. The development of FAIMS science since the introduction of these ion types in early work by Guevremont has brought forward a deeper understanding and re‐evaluation of these definitions in several aspects. First, all “ion types” are actually “ion‐gas pair” types—the same ions can assume different types in different gases based upon the ion/molecule interactions between these species (Barnett et al., 2000; Levin et al., 2006a; Campbell, Zhu, & Hopkins, 2014; Liu et al., 2015; Liu et al., 2017). Generally, increasing the mass and polarizability of gas molecules shifts ions from type C to B to A. Hence, the ion type should be reported only with reference to the gas as “species X^+^ has behaved as a type C ion in N_2_.” Second, since all type A ions will eventually turn into type B at higher *E*_D_ (Shvartsburg, 2009; Shvartsburg et al., 2009), the existence of type A ions is an artifact of *E*_D_ range limited by hardware (arc breakdown in gas or waveform generator constraints), fragmentation of precursor or other loss (e.g., scattering), or just explored experimental range. Hence, the type A should be reported only in conjunction with the covered *E*_D_ (or *E*_D_/*N*) range as “species X^+^ has behaved as type A ion in N_2_ up to *E*_D_ of 30 kV/cm explored here.”

Similarly, the conversion of type C ions to a new type (*E*_C_ become more positive with increasing absolute *E*_D_ up to certain point, then less positive at higher *E*_D_) was recently encountered in He/N_2_ mixtures with high He content (Shvartsburg, Ibrahim, & Smith, 2014) and, by analogy to type A transitioning to type B, may be termed type D. This type appears much less common than B, as all type A ions must turn into B but not all type C ions turn into D. Possibly type D only occurs in certain gas mixtures due to non‐Blanc (Biondi & Chanin, 1961) effects.

Finally, large macromolecular ions (including proteins above ∼30 kDa) exhibit extremely negative *E*_C_ at high *E*_D_ (Shvartsburg et al., 2009). This effect must be due to electric dipole alignment during the high‐field waveform segment dramatically raising the *K* value in that segment relative to low‐field segments with weaker or no alignment. While this behavior superficially resembles the type D, it manifests much more dramatically with *E*_C_ becoming negative (not just decreasing from a maximum), has unrelated physical cause, and emerges for much larger ions. Hence we propose assign such species to a new type E.

##### Relating ion types to *K* (*E*) functional forms

While the ion types were defined in terms of *E*_C_ trends depending on *E*_D_, they were related to *K*(*E*) forms. The types A and C correspond to *K* increasing and decreasing as a function of *E* or *E*/*N*, respectively. The type B was ascribed to *K* rising to a maximum and then dropping at higher *E*. That is not necessarily true: a *K*(*E*) function that continues increasing with decreasing slope (i.e., negative second derivative) can yield type B behavior as the absolute difference between *K* values at two *E*_D_ with fixed ratio decreases (Shvartsburg, 2009). Similarly, a *K*(*E*) function that continues decreasing with decreasing slope (i.e., positive second derivative) can yield type D behavior as the absolute difference between *K* at two *E*_D_ with fixed ratio decreases. We advise making no conclusions about the *K* (*E*) functions for type B and D ions, unless upon quantitative extraction as discussed above.

### E. Determination of *K*_0_ (Step 5) and *CCS* (Step 6)

#### 1. Definition of the Representative *X* From a Distribution of *X*

The primary measurable *X* is often the arrival time *t*_A_, and the distribution is the ATD. The first step is to determine the centroid arrival time for the ion population of interest, at each voltage. A comprehensive report should document (i) the definition of the centroid (e.g., apex, center of a Gaussian,…), (ii) the procedure (e.g., smoothing, fitting by a specified function, fixed parameters such as peak width and justification of the choice,…), (iii) the software or script used (name, version, provider) and (iv) the resulting centroid values and their uncertainties.

#### 2. Primary (DTIMS) Method

Even when the final aim is to obtain *CCS* values, we recommend that *K*_0_ values are also explicitly included in the data report. The reasons are: (1) instrument calibrations should be carried out in the *K*_0_ domain to avoid undue extrapolation, (2) *K*_0_ values (but not CCS) can be determined in gas mixtures without introducing error, (3) the combined uncertainty of *CCS* differs from that of *K*_0_.

##### *K*_0_ values

Most instruments measure not directly the time the ions take to traverse the drift region (*t*_d_), but the arrival time *t*_A_, which is the sum of *t*_d_ plus a time *t*_0_ spent outside the drift region. Multi‐field experiments involve measuring arrival times as a function of the inverse drift voltage, making a linear regression, and obtaining *t*_0_ from the intercept and *K*_0_ from the slope (Equation (7)). The procedure thus assumes that *K*_0_ does not change with *E*/*N* over the range of fields defined by the different Δ*V* values. Some bias (thus, uncertainty) is associated with this assumption.

(7) $$t_{A}=t_{d}+t_{0}=\left(\frac{L^{2}}{K_{0}}\cdot \frac{T_{0}p}{{Tp}_{0}}\right)\cdot \frac{1}{\Delta V}+t_{0}=\text{slope}\cdot \frac{1}{\Delta V}+t_{0}$$

The software and procedure used for the linear regression should be specified and the uncertainty of the intercept and slope (and how it was determined) should be documented. Note that a regression by least squares minimization correctly estimates the uncertainty of the slope if the data points are equally spaced on the *x*‐axis and if the variances are similar over the entire range. In that sense, the linear regression procedure itself contributes some uncertainty.

In addition to a graph of the linear regression and indication of its quality (e.g., *via r* ^2^), providing a plot of residuals can be informative: curvature may indeed indicate either that effective drift length varies as a function of the field, or that the mobility depends on *E*/*N* in the measurement range (Kemper, Dupuis, & Bowers, 2009; Allen & Bush, 2016; Hauck et al., 2017b).

##### Uncertainty of *K*_0_

There are different ways to convey uncertainty. The principle is to report what was done, how it is justified, and what it means. According to the GUM (JCGM, 2008), uncertainties should be provided in terms of standard uncertainties *u*, optionally accompanied with an expanded uncertainty *U*_p_ associated to a given level of confidence (e.g., the range including a percentage *p* of the distribution), the associated coverage factor *k*_p_ (*U*_p_ = *k*_p_.*u*), and its justification. Nonrestrictive examples are given below.

###### Propagation of uncertainty on *K*_0_ from the linear regression

The combined standard uncertainty *u*_c_ of each *K*_0_ value can be estimated from the propagation of the standard uncertainties *u* of each component (*L*, *p* and *T* from the experimental parameters in step 3, standard error of the *slope* from the linear regression in step 5), which is given by Equation (8), assuming that the uncertainties are uncorrelated:

(8) $$u_{c}\left(K_{0}\right)=K_{0}\cdot \sqrt{{\left(2\frac{u\left(L\right)}{L}\right)}^{2}+{\left(\frac{u\left(T\right)}{T}\right)}^{2}+{\left(\frac{u\left(p\right)}{p}\right)}^{2}+{\left(\frac{u\left(\text{slope}\right)}{\text{slope}}\right)}^{2}}$$

This uncertainty evaluation is quick, and takes into account how *K*_0_ may fluctuate with Δ*V*, than thus with *E*/*N* over the chosen field range. Usually, the contributions of the uncertainties of *T* and *p* dominate the combined uncertainty. Whether the uncertainty of the slope takes into account the uncertainties of *t*_A_ and Δ*V* depends on how it was determined (i.e., whether the linear regression software uses the data points only, or whether the regression parameters and their uncertainty was weighted using vertical and horizontal error bars on each data point).

Other methods of uncertainty evaluation exist, such as Monte Carlo simulations. With that approach, the relative standard uncertainty of the drift time (hence, on 1/*K*_0_ or on *CCS*) was estimated at 0.27% on an Agilent 6560 IMS‐Q‐TOF modified by the manufacturer to make more accurate measurements (Stow et al., 2017). With that instrument, ^DT^ *CCS*_N2_ values for the Agilent tunemix ions in positive and negative mode were determined (Stow et al., 2017).

###### Standard deviation from technical replicates (independently repeated experiments)

The entire procedure, including the gas equilibration in the tube, should be replicated. This standard deviation thus gives a sense of the precision under repeatability conditions. The standard deviation (*u*) and number of independent replicates (*n*) should be mentioned. For data intended as primary standards, we recommend *n* ≥ 6, in order to minimize the expanded uncertainty for a certain level of confidence. When reporting average values from multiple independent measurements, in case a quality criterion (e.g., *r* ^2^ or residuals of the linear fit) was used to accept/reject values, it should be specified.

###### Estimation of the uncertainty associated with a single measurement

For rare samples, replication is not always possible. One may then (i) propagate uncertainty from the linear regression; (ii) use knowledge of uncertainties determined previously for similar analytes with the same procedure in the same lab; (iii) use published values of uncertainties evaluated thoroughly with the same instrument model, with justification of how close the procedures are (e.g., use the interlaboratory precision values reported for the step‐field procedure on the Agilent 6560 IMS‐Q‐TOF (Stow et al., 2017)); or (iv) invoke the conservative value of 0.8% s.d. discussed in section IV.A for previously published values coming from different DTIMS instrument designs. All are acceptable if properly justified so that the meaning is clear to the reader.

##### CCS values and their uncertainties

Combining the expression of *K*_0_ coming from the slope with Equation (4) gives:

(9) $$\text{slope=}\frac{16}{3}\sqrt{\frac{k_{B}}{2\pi }}\cdot \frac{N_{0}T_{0}}{p_{0}}\cdot L^{2}\cdot \frac{p}{\sqrt{T}}\cdot \frac{\text{CCS}\cdot \sqrt{\mu }}{z}$$

The combined uncertainty on the CCS determined from a single experiment (single regression) is:

(10) $$u_{c}\left(\text{CCS}\right)=\text{CCS}\cdot \sqrt{{\left(2\frac{u\left(L\right)}{L}\right)}^{2}+{\left(0.5\frac{u\left(T\right)}{T}\right)}^{2}+{\left(\frac{u\left(p\right)}{p}\right)}^{2}+{\left(\frac{u\left(\text{slope}\right)}{\text{slope}}\right)}^{2}+{\left(\frac{u\left(z\right)}{z}\right)}^{2}+{\left(\frac{u\left(\mu \right)}{\mu }\right)}^{2}}$$

Compared to *K*_0_, the temperature weights differently on the uncertainty. Simple propagation of error from *K*_0_ would ignore the (negative) correlation between the temperature effects. As for *z* and *μ*, the peak assignment (step 4) plays a role. If one assumes that the uncertainty of the ion charge is zero, proper justification should be given. Note that automatic assignment of charge states may fail in some cases, especially as *z* becomes very large. One often assumes that the relative error of *μ* is negligible, but a word of caution is warranted. Indeed the ion mass *m*_i_ (provided that *z* is certain) and gas mass *m_g_* can be known with a very high precision and accuracy, but the gas purity plays a role here: traces of other gases will also contribute to an uncertainty of *μ*. Note that this effect involves not only the purity of the gas intentionally introduced inside the IMS, but also the nature of the gases present outside the IMS and leaking inside the tube. These effects may be far from negligible, and may contribute significantly to the differences observed with different drift tube designs (see section IV.A). This is the main reason why not only the purity of the gas introduced in the drift tube, but also all information on pressure differentials between the tube and neighboring zones, especially if the nature of the gas in the neighboring zone(s) is(are) different, should be included in the experimental details of step 3. In a similar way as for *K*_0_, repeated experiments give a sense of the precision under repeatability conditions. Again, the whole procedure has to be repeated, including gas equilibration. This is a better way than (10) to account for fluctuations in gas purity.

If any equation other than Equation (4) is used, the mathematical function and all input parameters should be fully documented based on the same principles: the users should have all information to link *CCS* and *K*_0_ values and calculate the uncertainties.

#### 3. Secondary Methods: General Recommendations

##### Choice of calibrants

DTIMS (with either direct *t*_d_ measurement or the step‐field method) is the only primary method to measure for *K*_0_ values and the experimentally derived CCS values. Accordingly, the uncertainty of CCS values, derived by this method for use as calibrants needs to be carefully evaluated. Uncertainty estimation of calibrant ions should be performed following the principles given in state‐of‐the‐art documents as the ISO Guide for the Expression of Uncertainty in Measurement (JCGM, 2008) and QUAM (EURACHEM/CITAC, 2012), with examples provided in the previous section. The reports for secondary methods should thus include:

– the list of calibrant ions, with motivation of the choice (specific recommendation for the different methods will be given in the following sections),
– the ^DT^ *K*_0_ or ^DT^ *CCS* values used for calibration (e.g., from references to the primary literature), with specification of the gas and temperature at which these were determined,
– the uncertainties attributed to these ^DT^ *K*_0_ or ^DT^ *CCS* values (if not provided according to the recommendations of section V.E.2, see section IV.A), either expressed as standard uncertainties or as 95% confidence interval. Obviously the uncertainty of the results obtained following calibration.
– documentation of steps 1–4 for the calibrants.

##### Calibration curves

**Figure 4:**
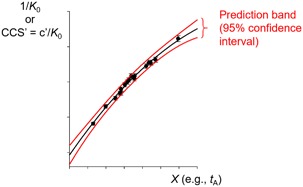
Exemplary calibration curve (measured X as *x*‐axis, calibrant inverse mobility or parameter proportional thereto as *y*‐axis), wherein the standard uncertainty of the mobility of the calibrants is displayed as error bar. The prediction band gives the interval in which there is 95% likelihood to make one or more future observations.

The calibration gives the instrumental function that relates the measurable *X* to *K*_0_. Alternatively, a parameter directly proportional to *K*_0_ (the proportionality factor is thus assumed to be the same for all calibrants and analytes) can be calculated from ^DT^ *CCS* values and used instead of *K*_0_. If the analyst intends to assess and report the uncertainty of measurement following the (so‐called bottom‐up) procedure outlined in the ISO Guide for the Expression of Uncertainty in Measurement and in QUAM (Chapter 4), the uncertainty of CCS calibrants is an important component contributing to the total combined uncertainty of the measurand (see Fig. 4). The European Accreditation document EA‐4/02M: 2013 gives additional guidance and examples specifically for calibration (EA, 2013). If the measurement uncertainty is derived from experimental data (i.e., from in house validation studies, collaborative method validation studies, or from QC data), the influence of calibrant uncertainty (i.e., the precision of slope and intercept) can be determined by multiple independent replicates (see VIM (JCGM, 2012) for definition of precision).

We recommend showing representative calibration curves, and providing the calibration equation(s) relating *X* to *K*_0_ (typical equations for the different methods will be given in the following sections). To estimate the value of the analyte, it does not matter whether the *x*‐axis is *X* or *K*_0_. However, to estimate the uncertainty of the value, the measured value *X* should be on the *x*‐axis and the calibration/predicted value *K*_0_ (or the value proportional to *K*_0_) on the *y*‐axis, because regression analysis is designed to determine *y* from a given *x*, and several software packages can provide prediction bands alongside with predicted values. Such prediction bands take into account the number of degrees of freedom of the calibration curve (number of calibration points minus the number of fitted parameters; the prediction band narrows when the number of degrees of freedom increases) and the spread of the calibration points. Prediction bands are very useful to compare the figures of merit of different calibration strategies (Haler et al., 2017). Further, although it is not yet common practice, it would be useful to display the uncertainty of both *X* and *K*_0_ (or the value proportional to *K*_0_) as error bars, and to use a software able to weigh the regression and its prediction band according to these error bars.

##### Quality control after calibration

Calibration is a well‐known analytical procedure and, for CCS measurements, standard quality control schemes and measures can be applied. Accordingly, we also recommend including QC injections after the calibration process, during the sequence of experiments. The QC should be an independent material (i.e., one not used to calibrate the instrument at the time of the experiment). This QC is to check the trueness of the experimental CCS regarding the calibration procedure. Replicates of this(ese) QC point(s) should also provide a good estimate of the precision under repeatability conditions of measurement, which can be used to gauge that of the experimentally determined CCS.

#### 4. DTIMS Single‐Field Experiments

##### Procedure

In DTIMS, instead of the multi‐field primary measurement method, a convenient method (especially for LC‐MS) is to use a single field strength for measurements of both established calibrant ions and unknown ions (Mordehai et al., 2015; Stow et al., 2017). To find the relationship between the measured arrival time *t*_A_ and *K*_0_, one starts from Equation (7), and the expression of 1/*K*_0_ as a function of *CCS* (Equation (11)).

(11) $$\frac{1}{K_{0}}=\frac{16N_{0}}{3}\sqrt{\frac{k_{B}T}{2\pi }}\cdot \;\;\text{CCS}\cdot \frac{\sqrt{\mu }}{z}$$

*t*_0_ is assumed to be the sum of a fixed term *t*_fix_ and a mobility‐dependent term:

(12) $$t_{0}=t_{\text{fix}}+\beta_{1}\cdot \gamma \;\text{CCS}$$

where *γ* =  [*m_i_*/(*m_g_* + *m_i_*)]^1/2^/*z*, to be calculated for each calibrant and analyte ion, and *β*_1_ is a constant derived from Equation (4) depending on the drift gas employed and the ion transfer settings outside of the drift region. By replacing in Equation (7), one obtains:

(13) $$t_{A}=t_{\text{fix}}+\beta_{1}\cdot \gamma \;\text{CCS}+\beta_{2}\cdot \gamma \;\;\;\text{CCS}\text{=}t_{\text{fix}}+\beta \cdot \gamma \;\text{CCS}$$

where $\beta_{2}\cdot \gamma \;\;\;\text{CCS}$ is the true drift time (*t*_d_) derived from Equation (4), and *β*_2_ is a coefficient dependent on the experimental gas pressure, temperature, electric field, and geometry of the drift cell. The term *β* thus reflects the aggregate of experimental conditions experienced by the ion (i.e., temperature, pressure, field strength) and is directly taken from the slope of a calibration curve of γ×^DT^ *CCS* values for reference calibrant ions. This is a linear function defined by two parameters, *β* and *t*_fix_.

The underlying assumptions are thus:

– that *t*_fix_ is the same for the calibrant and analytes (i.e., that *m/z* dependent, TOF‐like effects are negligible (Mordehai et al., 2015; Stow et al., 2017)),
– that the *CCS* is the same inside and outside the drift region, and thus equal to the ^DT^ *CCS* measured by the step‐field method. Most of the drift contributing to *t*_0_ occurs in the ion funnel containing the same gas as the drift region, but drift occurs in the collision cell as well. Small analyte‐dependent effects on *t*_0_ were found when a different gas was used in the collision cell compared to the drift region (Marchand et al., 2017). If the same gas is used (typically N_2_), the calibrant and analyte need not be of the same chemical class.

The calibrant and analyte have to be acquired with the same IM settings (so that *β*_2_ is constant) and the same MS settings (so that *β*_1_ and *t*_fix_ are constant). Also, if the drift gas and its temperature are the same as in the primary method and the *E*/*N* falls within the range used in the primary method, the measurand has the same definition as in the primary method. If, for example, the gas or temperature differ, the single‐field method becomes an empirical method.

Routine single‐field operation should report key experimental IM‐MS variables (drift gas, temperature, pressure, field strength, calibrant ions, and MS voltages that can affect *t*_fix_ or *β*_1_), which can assist in assessing potential analyte dependencies on such factors. Ideally, routine collection of single‐field data needs to be controlled in terms of accuracy by comparison to multi‐field results utilizing independent (non‐calibrant) compound sets.

##### Evaluation of uncertainty

The nature of the calibrant ions (i.e., molecular class, charge state) does not need to match that of the unknown ions, but precise and reproducible measurement conditions as well as agreement on a unified single‐field calibration approach are critical for providing the most accurate ^DT^CCS values with the single‐field method. A recent interlaboratory study (Stow et al., 2017) demonstrated extremely low bias between multi‐ and single‐field measurement modes on the same instrument platform (average absolute bias of 0.54%, details per chemical class can be found in the paper (Stow et al., 2017)) for more than 90 ion species. This was achieved by ensuring inter‐instrument compliance regarding the ^DT^ *CCS* values of the calibrant set determined in the multi‐field approach.

If using different conditions (empirical method), the bias should be studied for some primary standards to evaluate the uncertainty.

#### 5. TWIMS

##### Procedure

Currently, CCS values cannot be derived from first principles using the TWIMS device, but must be obtained following calibration with standards of known CCS value, primarily derived from DTIMS measurements. To generate ^TW^CCS values, standards of known ^DT^CCS value must be measured to generate a calibration curve (Ruotolo et al., 2008; Smith et al., 2009; Thalassinos et al., 2009; Bush et al., 2010). Published ^DT^CCS values (He or N_2_) are converted to a value *CCS*’, which according to Equation (4) is proportional to 1/*K*_0_:

(14) $${\text{CCS}}^{\prime }=\frac{\text{CCS}\sqrt{\mu }}{z}=\frac{3}{16}\sqrt{\frac{2\pi }{k_{b}T_{\text{eff}}}}\frac{e}{N_{0}K_{0}}=R\frac{1}{K_{0}}$$

Whilst the neutral gas in TWIM cells (for Synapt G2 and later) is a mixture of He and N_2_, CCS values measured for same species are highly correlated across gasses (Bush, Campuzano, & Robinson, 2012). Helium reference values have been used previously to calibrate N_2_ measurements (i.e., ^TW^ *CCS*_N2‐>He_ experiments), and the discrepancies introduced by measuring in N_2_ with a limited admixture of He will be proportionally smaller. Direct comparisons of CCS values measured on various DTIM and TWIM instruments (Giles et al., 2015) show that Synapt G2‐Si data fall within the range obtained using other instruments.

The instrumental constant *R* contains the effective temperature of the ion‐buffer gas collisions. Then the following procedure assumes same *R* for the calibrant ions and for the ion of interest. This assumption warrants discussion. The effective temperature *T*_eff_ is equal to the bath gas temperature *T*_gas_ only in the low‐field limit. According to the two‐temperature treatment, *T*_eff_ = *T*_gas_ + *m*_gas_ *v_d_* ^2^/3*k*_B_ for non‐negligible but low *E*/*N* (Siems, Viehland, & Hill, 2016)_._ However, in TWIMS the field applied to the ions is changing over time, and Equation (1) may not be satisfied at all times because of velocity relaxation effects (the ions need time to reach the steady state defined by Equation (1)) (Richardson, Langridge, & Giles, 2018). Empirically, effective ion temperatures were found to be significantly higher than bath gas temperatures (Morsa, Gabelica, & De Pauw, 2011; Morsa, Gabelica, & De Pauw, 2014), so these effects are significant. Thus the necessity for *R* to be the same for the calibrant and analyte is one of the reasons why chemical classes and charge states must be matched. The magnitude of the factor needed to correct for this calibrant‐related bias has not yet been established, and progress is expected in that direction. Meanwhile, it is crucial to report the full list of calibrant ions and their associated ^DT^ *CCS* values used for the calibration. *CCS*′ is plotted as a function of *t′*, defined by

(15) $$t^{\prime }=t_{A}-c\sqrt{\frac{m}{z}}$$

where *c* is a constant depending on the voltages used in the transfer optics (obtained from the MassLynx software; note that the resulting corrections are typically of the order of 100 µs, compared to measured drift times of several ms, and uncertainties in the determination of *c* therefore have a small effect on the final measurement). The derived CCS′ versus *t*′ data are fitted using an empirically determined power law (Wildgoose et al., 2006),

(16) $${\text{CCS}}^{\prime }=A\;{t^{\prime }}^{N}$$

its linearized version (Ruotolo et al., 2008),

(17) $${{\text{ln}}_{}}_{}CCS=\text{ln}{}_{}\;A+N\times \text{ln}{}_{}t$$

or a polynomial (Bush, Campuzano, & Robinson, 2012):

(18) $${\text{CCS}}^{\prime }=A\;t^{\prime 2}+B\;t^{\prime }+C\;$$

The calibration curve derived in this way is subsequently used to calculate the ^TW^CCS of the ions of interest from their measured *t* values, obtained under identical operating conditions to those of the calibrant species. An undetermined time offset, *t*_0_, intended to capture any additional timing delays in the system (assumed constant for all ions) may also be introduced into the calibration for improved accuracy. The power law calibration then becomes:

(19) $${\text{CCS}}^{\prime }=A{\left(t^{\prime }-t_{0}\right)}^{N}$$

Equation (19) is currently adopted by Waters to calibrate data from the Synapt G2‐S, G2‐Si and VION instruments using the MassLynx/UNIFI software platform.

##### Calibrant choice for TWIMS

For current TW calibration procedures, matching the charge state (singly versus multiply charged) and chemical class of calibrant provides calibrated data that agree better with drift tube data (Thalassinos et al., 2009; Bush, Campuzano, & Robinson, 2012; Wright et al., 2013; Gelb et al., 2014). TWIMS is an empirical method relying on calibration because at this point in time it not fully understood how to quantitatively account for both the velocity relaxation and the oscillatory field for all ion types. For TIMS, which operates at similar *E*/*N* to TWIMS but with non‐oscillatory fields, the compound class has no influence on the calibration at the current level of precision, so the compound‐class effects in TWIMS are most probably due to velocity relaxation. Compound class matching for similar velocity relaxation effects means that the analyte and calibrant should have similar *K*_0_ = *f*(*m*/*z*) dependencies (Richardson, Langridge, & Giles, 2018), and these dependencies segregate with the compound class (lipids, peptides, carbohydrates, nucleic acids, folded proteins, unfolded proteins,…) (McLean, 2009). In the future, the calibration method may be generalized to explicitly account for velocity relaxation (Richardson, Langridge, & Giles, 2018). Further, using ^DT^CCS_He_ values to calibrate TWIMS instrument working mainly in nitrogen (which was frequent in the early days, as often only ^DT^ *CCS*_He_ values were available) adds other compound‐class effects (Gabelica & Marklund, 2018) related to the interaction potentials of nitrogen and helium with the exposed surface groups (Bleiholder et al., 2015). Here, the charge density will play the largest role (Young & Bleiholder, 2017), and for multiply charged proteins these effects become significant (Canzani, Laszlo, & Bush, 2018) compared with measurement precision. For small molecules, dipole moments play a much larger role in N_2_ than in He (Warnke et al., 2015), and it is thus recommended to use ^DT^ *CCS*_N2_ values to calibrate TWIMS.

Some groups have proposed using calculated CCS values to calibrate TWIMS because ^DT^ *CCS* values were unavailable for their molecular classes (phosphoric acid clusters (Lavanant, Tognetti, & Afonso, 2014), large protein complexes (Marklund et al., 2015), nucleic acids (Lippens et al., 2016)). However, in the framework of measurement sciences, calculating theoretical CCS values is not equivalent to an independent primary method for ion mobility measurements for three main reasons. First, ion mobility spectrometry primarily measures mobilities (*K*, in m^2^ V^−1^ s^−1^), not surfaces (*CCS*, in m^2^). Collision cross section values are not directly traceable to the SI (the meter). Second, the uncertainty is higher, and a large component of it comes from uncertainties about the gas‐phase geometries assumed by the ions. Third, even in the most physically accurate calculation method (trajectory model), the Lennard‐Jones parameters have been themselves parameterized against DTIM measurements (e.g., for carbon, using values measured for C_60_^+^) (Mesleh et al., 1996), so the method is not independent. Thus, calculated values cannot be traced to the international system of units, and using calculated values for calibrating instruments contradicts with the fundamental principles of metrology. What is encouraged is to collect more high‐quality ^DT,1ry^ *CCS* data.

In summary, empirical methods, by definition, assume that bias (if the bias was known, it should be corrected for). Thus the result will depend on the choice of the calibrants. Thus, given the empirical nature of TWIMS, it is of utmost importance to provide the full list of calibrant ions and the values used for the calibrants.

#### 6. TIMS

The separation in a TIMS device can be described in the center of mass frame, and is therefore based on the same principles as in DTIMS. This separation depends on the bath gas flow velocity (*v_g_*), and ion confinement and elution parameters. The primary measurand in a TIMS experiment is the scan number, which is related to the ion trapping and elution voltage parameters. Absolute ion mobilities *K*_0_ or collision cross sections of the analyte ions are currently not accessible directly, but determined by a calibration procedure with standards of known mobilities. The calibration can be performed internally or externally.

TIMS separation depends on *v_g_*, elution voltage (*V_e_*), ramp time (*t_ramp_*), and base voltage (*V_out_*). The reduced mobility, *K_0_*, is defined by,

(20) $$K_{0}=\frac{v_{g}}{E}≅\frac{A}{\left(V_{e}-V_{out}\right)}$$

where *A* is an instrumental constant. Three different calibration procedures have been described.

##### Calibration from experiments at different scan rates

Running the TIMS experiment at different scan rates allows one to determine the time outside of the TIMS cell (*t*_0_), followed by the determination of the elution voltage and the calibration constant (Hernandez et al., 2014). The total analysis time (t_Total_) is given by:

(21) $$t_{total}=t_{trap}+\left(\frac{V_{e}}{V_{ramp}}\right)t_{ramp}+tof=t_{0}+\left(\frac{V_{e}}{V_{ramp}}\right)t_{ramp}$$

where, *t_trap_* is the thermalization/trapping time, *tof* is the time after the ion mobility separation, and *V_ramp_* and *t_ramp_* are the voltage range and time required to scan that range, respectively. The elution voltage (*V_e_*) is experimentally determined by varying the ramp time for a constant ramp voltage. A linear dependence of *t_total_* on *t_ramp_* for all the investigated *m/z* is obtained. From the slope and the intercept of this graph, the *t*_0_ and *V*_e_ can be determined for each *m*/*z* range of interest.

##### First order calibration

Alternatively, a first order calibration is possible for an acquisition at a single scan rate, using known mobility peaks. This approach is based on the emprical observation that the “elution voltage” *V*_e_ is tightly correlated to the reduced mobility of the ions (Silveira, Ridgeway, & Park, 2014; Liu et al., 2018). This approach employs the following empirical calibration function:

(22) $$K_{0}=a+b\left(\frac{1}{V_{e}}\right)$$

where *a* and *b* are calibration constants and the elution voltage *V*_e_ is approximately the voltage across the TIMS analyzer. In commercial versions of the software, the calibration from scan number to 1/*K*_0_ is performed automatically using a linear regression of *K*_0_ as a function of 1/*V*_e_. This approximation assumes an average *tof*.

Equation (22) has been successfully used to calibrate TIMS spectra. For example, cross sections obtained for ubiquitin (Liu, Kirk, & Bleiholder, 2016) or cytochrome *c* (Molano‐Arevalo et al., 2017) agree well with the drift tube values (Bleiholder et al., 2015). More recently, a tandem‐TIMS device was constructed from coupling two TIMS analyzers. The tandem‐TIMS spectra can also be calibrated according to Equation (22) (Liu et al., 2018). Cross sections determined for ubiquitin closely match the drift tube values. Overall, this calibration scheme reproduces drift tube ion mobilities within 1% (Chai et al., 2018).

Importantly, the constants *a* and *b* depend strongly, and in an unknown manner, on the TIMS operating settings. This is a consequence of the empirical nature of the calibration function. Hence, the calibrated coefficients are only valid for the exact TIMS settings (scan rate, range and pressure) used for their determination (Chai et al., 2018).

##### Transferrable calibration

A transferable and sample‐independent calibration procedure was recently reported for TIMS based on a Taylor expansion of instrument properties (TEIP) (Chai et al., 2018). This is based on a calibration function derived from a solution to the Boltzmann transport equation, instead of from empirical correlations. TEIP then separates the quantities depending on the sample (*m*/*z*, *K*_0_) from those depending on the TIMS instrument (*β*, *v*_g_, etc). Finally, a Taylor‐expansion is performed for the instrument‐dependent quantities, and the resulting coefficients form the basis for the coefficients. These calibration coefficients were found, to a good approximation, constant over time and independent of instrument settings or sample properties (Chai et al., 2018).

##### Calibrants for TIMS

The only significant difference between the definitions of the measurand in TIMS and DTIMS is the magnitude of *E*/*N*. Currently, *E*/*N* in TIMS is assumed low enough that these definitions are equivalent. The level of measurement accuracy needed to invalidate this assumption is not yet documented; currently the assumption seems valid. Thus, a single compound class is assumed to suffice for calibration. Commercial instruments use the Agilent tuning mixture as calibrants. However, the values currently embedded in the calibration files (Chai et al., 2018) differ by 0.75% on average from those reported from DTIMS (Stow et al., 2017) and used to calibrate the single‐field measurement in Agilent instruments. Thus, a significant bias between two methods (DTIMS vs. TIMS) is directly attributable to the choice of primary values for the calibrant ions, rather than the measurement principle or instrument design. This illustrates how important and urgent it is for the community to agree on values for a set of primary standards.

#### 7. DMA

Absolute *K*_0_ determination is possible by precise measurement of the sheath flow rate, gas composition, temperature, pressure, and DMA geometry (with uncertainties reported). However, as noted above, it is often much easier to proceed *via* a calibration using compounds with *K*_0_ determined previously with low uncertainty. While the former is preferred, the latter is commonly utilized for DMA measurements made in environmental settings, where instruments are operated remotely and broad mobility distributions are of interest. Tetra‐alkylammonium ions are popular calibrants for DMA (Ude & Fernández de la Mora, 2005).

With known calibration, for a fixed sheath flow rate, a plot of the known inverse mobilities versus the applied voltage where the ion is maximally transmitted should be linear (for all DMA geometries), which can be used to subsequently link applied voltage to mobility.

## VI. CONCLUSIONS AND OUTLOOK

We presented a concerted effort to outline the fundamental principles underlying ion mobility measurements, aiming to reduce the confusion among users. Reflecting on how to apply the GUM (JCGM, 2008) and QUAM (EURACHEM/CITAC, 2012) guidelines to ion mobility measurements gives important insight about (i) the definition of the measurand (what we measure in ion mobility); (ii) the distinction between primary methods of measurements and secondary or empirical methods; and (iii) the sources of uncertainty of ion mobility measurements.

Ion mobility spectrometry measures the ion mobilities (*K*), not surfaces (*CCS*). Reduced mobility (*K*_0_) and CCS values depend solely on the ion structure, the gas nature, the temperature, *E*/*N*, but not *per se* on the method of measurement. However, a distinction must be made between a primary method of measurement (DTIMS), wherein the mobilities are traceable to the SI, and secondary methods, wherein the mobilities are traceable to those of the reference compounds used for calibration. Here we provide recommendations to make this traceability possible.

We also highlight the difficulties and confusion caused by the absence of commonly agreed upon reference materials and *consensus* values for primary standards for ion mobility spectrometry. Several sets of compounds and values currently coexist. This situation is akin to that of mass spectrometry before the agreement on the mass of carbon 12 (Cameron & Wichers, 1962). Progress toward agreeing on values of the highest metrological quality for primary standards is urgent, to fully take advantage of the recent advances in precision of ion mobility measurements. To determine the best standards and their values, the community would benefit from a round robin study using exclusively the primary method, comparing values obtained with drift tube instruments having different designs. These standards and their reference values shall eventually be used to assess the merits of secondary methods of measurements or compare the merits of different calibration procedures. Meanwhile, when comparing secondary methods or calibration procedures, the study design should eliminate as far as possible the variabilities related to the effects of ion structures and of unrecognized biases in the calibration inputs.

Although the present article was conceived as a guide and provides recommendations rather than strict guidelines, one may need such guidelines in the future, for example to establish data standards, reporting standards, data processing software packages, data formats and databases. Common databases of *CCS* and *K*_0_ would need to include all information about the methods used for data acquisition (steps 1–3) and processing (steps 4–6) to obtain the values and their associated uncertainties. The present recommendations should constitute the foundation for future guidelines and standards in ion mobility.

## CONFLICT OF INTEREST DISCLOSURE

Valérie Gabelica, Carlos Afonso, Perdita Barran, Justin L. P. Benesch, Christian Bleiholder, Michael T. Bowers, Aivett Bilbao, Matthew F. Bush, Iain D. G. Campuzano, Colin S. Creaser, Edwin De Pauw, Johann Far, Francisco Fernandez‐Lima, Hugh I. Kim, Kevin Pagel, Frédéric Rosu, Frank Sobott, Konstantinos Thalassinos and Thomas Wyttenbach declare no conflicts of interest. Alexandre A. Shvartsburg has interest in Heartland MS that provides the ion funnel and FAIMS systems for various mass spectrometers (including those mentioned in the article) and receives royalties from Battelle on licensed IP implemented in commercial IMS/MS instruments (including the Agilent 6560 and Bruker timsTOF mentioned in the article). He also holds a faculty appointment at the Moscow Engineering Physics Institute (MEPhI), Russia. Tim Causon and Stephan Hann are recipients of a University Relations Grant from Agilent Technologies (#4240). Brian H. Clowers receives royalties from licensed intellectual property held by Battelle Memorial Institute (US7888635B2), which is used in commercial IMS/MS instruments. Christopher J. Hogan Jr. receives royalties on a commericial IMS (Kanomax‐FMT) coupled to a condensation particle counter for nanoparticle and macromolecular analyte detection. Drs. John A. McLean and Jody C. May have collaborative agreements in place with Agilent Technologies (Santa Clara, CA) and Waters Corporation (Milford, MA). The Center for Innovative Technology at Vanderbilt University is designated a Waters Center of Innovation and acknowledge financial support in the form of an Agilent Thought Leader Award. Stephen J. Valentine receives royalties from Waters for patents related to the IMS‐TOF technology. J. Larry Campbell is an employee of SCIEX, a manufacturer of Differential Mobility Spectrometry (DMS) technology. K Giles and K Richardson are employees of Waters Corporation, Wilmslow UK who design, develop and sell mass spectrometry instruments utilizing the traveling wave ion mobility separators discussed in this article. John C. Fjeldsted and Ruwan T. Kurulugama are employed by Agilent Technologies, a commercial supplier of Ion Mobility Mass Spectrometry instrumentation and informatics products. Mark E. Ridgeway is an employee of Bruker Daltonics, a manufacturer of TIMS ion mobility technology. Michael Groessl is consulting for Tofwerk, a commercial supplier of ion mobility mass spectrometry instrumentation.
